# Vat photopolymerization-based 3D printing of polymer nanocomposites: current trends and applications

**DOI:** 10.1039/d2ra06522c

**Published:** 2023-01-06

**Authors:** Mussadiq Shah, Abid Ullah, Kashif Azher, Asif Ur Rehman, Wang Juan, Nizami Aktürk, Celal Sami Tüfekci, Metin U. Salamci

**Affiliations:** a Additive Manufacturing Technologies Application and Research Center-EKTAM Ankara Turkey abid.ullah@gazi.edu.tr abidmech95@gmail.com; b Department of Mechanical Engineering, Faculty of Engineering, Gazi University Ankara Turkey; c State Key Laboratory for Mechanical Behavior of Materials, School of Materials Science and Engineering, Xi'an Jiaotong University P. R. China; d CAS Key Laboratory of Mechanical Behavior and Design of Materials, Department of Modern Mechanics, University of Science and Technology of China P. R China; e ERMAKSAN Bursa 16065 Turkey; f Department of Industrial Engineering, Nanchang Hangkong University Nanchang P. R China; g Advanced Manufacturing Technologies Center of Excellence-URTEMM Ankara Turkey

## Abstract

The synthesis and manufacturing of polymer nanocomposites have garnered interest in recent research and development because of their superiority compared to traditionally employed industrial materials. Specifically, polymer nanocomposites offer higher strength, stronger resistance to corrosion or erosion, adaptable production techniques, and lower costs. The vat photopolymerization (VPP) process is a group of additive manufacturing (AM) techniques that provide the benefit of relatively low cost, maximum flexibility, high accuracy, and complexity of the printed parts. In the past few years, there has been a rapid increase in the understanding of VPP-based processes, such as high-resolution AM methods to print intricate polymer parts. The synergistic integration of nanocomposites and VPP-based 3D printing processes has opened a gateway to the future and is soon expected to surpass traditional manufacturing techniques. This review aims to provide a theoretical background and the engineering capabilities of VPP with a focus on the polymerization of nanocomposite polymer resins. Specifically, the configuration, classification, and factors affecting VPP are summarized in detail. Furthermore, different challenges in the preparation of polymer nanocomposites are discussed together with their pre- and post-processing, where several constraints and limitations that hinder their printability and photo curability are critically discussed. The main focus is the applications of printed polymer nanocomposites and the enhancement in their properties such as mechanical, biomedical, thermal, electrical, and magnetic properties. Recent literature, mainly in the past three years, is critically discussed and the main contributing results in terms of applications are summarized in the form of tables. The goal of this work is to provide researchers with a comprehensive and updated understanding of the underlying difficulties and potential benefits of VPP-based 3D printing of polymer nanocomposites. It will also help readers to systematically reveal the research problems, gaps, challenges, and promising future directions related to polymer nanocomposites and VPP processes.

## Abstract 2

The synthesis and manufacturing of polymer nanocomposites have garnered interest in recent research and development because of their superiority compared to traditionally employed industrial materials.

## Graphical abstract

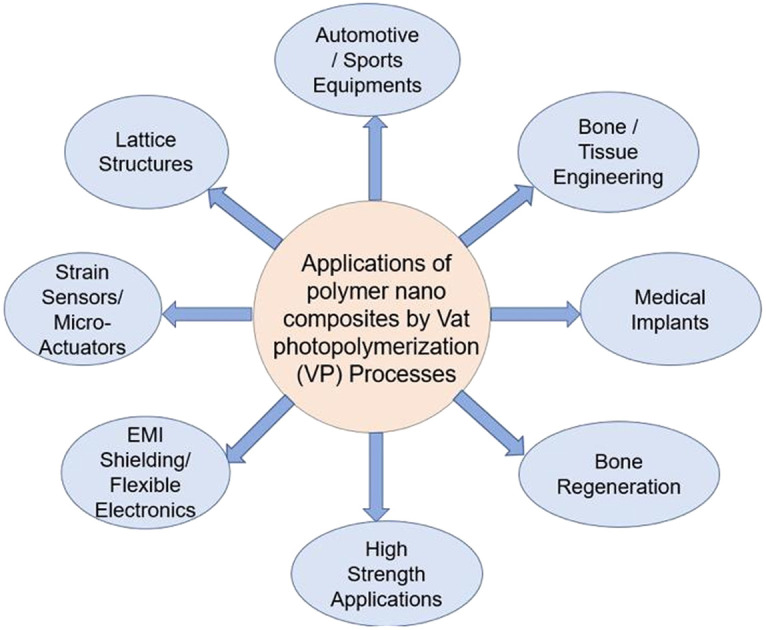

## 1. Introduction

Additive manufacturing (AM) is a group of manufacturing technologies that build objects in a layer-by-layer fashion from a CAD model. According to the ISO/ASTM 52900 standard, AM is defined as ‘‘the process of joining materials to make parts from 3D model data, usually layer upon layer, as opposed to subtractive and formative manufacturing methodologies’’.^1^ This technology was called rapid prototyping in the initial years, whereas nowadays the term 3D printing is widely used in a non-technical context, while the term additive manufacturing is used more formally.^1–3^ AM technologies can be grouped into seven different types, including material jetting, binder jetting, powder bed fusion, sheet lamination, material extrusion, direct energy deposition and vat photopolymerization.^4,5^ All these processes follow a common operation using the same steps, *i.e.*, joining 2d layers on top of each other until the 3D final part is built.^6^

AM has the ability to create complex parts in a short lead time, having great design freedom with little human interaction involved. AM as versatile technology has found applications in many fields such as aerospace, biomedical, automotive, energy and consumer products. AM is on the way to digitalization and will soon emerge as key technology in the next few decades in the context of Industry 4.0.^7,8^ The forecast for the next five years shows tremendous growth in the AM technology. According to Wohler's Report, the global revenue of all AM products and services grew by 19.5% to $15.2 billion.^9^ This is an increase from 7.5% growth in 2020, which was impacted greatly by the pandemic. The first commercialized method in AM is stereolithography (SLA), which was developed in 1988, followed by rapid advances in the next two decades, giving birth to other AM technologies. The past decade has witnessed significant research on VPP processes using nanoparticles to fine-tune the desired properties.

The use of polymer nanocomposites in VPP processes is a multidisciplinary area of research, which involves the interaction of machines, materials, computer control systems, *etc.* The synergy of both technologies showed tremendous enhancements in biomedical, structural, functional, electrical and thermal applications. If research and innovations continue at this pace, soon we will enter a new era of revolution where these processes can surpass the traditional manufacturing techniques.

However, not all research is focused on enhancing the desired properties, where technological aspects, filler matrix interfaces, design considerations, curing methods, *etc.* are also studied in parallel. There are several good in-depth reviews available. Readers who wish to study these detail are referred to the reviews regarding biomedical AM,^10–14^ current developments in AM,^15–18^ design for AM,^6,19–21^ materials in AM,^22^ polymers and polymerization,^23–31^ characterizations in AM,^32^ multi-material 3D printing,^33^ and nanocomposites in AM.^34–36^

Alternatively, in this review, we aim to provide a theoretical background and the engineering capabilities of VPP with a focus on the polymerization of nanocomposite polymer resins. Specifically, the configuration, classification and factors affecting VPP are summarized in detail. Furthermore, the different challenges in the preparation of polymer nanocomposites are discussed together with pre and post-processing. Subsequently, several constraints and limitations that hinder the printability and photo curability of nanocomposites are critically discussed. The main focus is the applications of printed polymer nanocomposites and the enhancement in their properties such as mechanical, biomedical, thermal, electrical and magnetic properties. The recent literature in the past three years is critically discussed and their main contributing results in terms of applications are summarized in the form of tables. Finally, the limitations, challenges, and possible approaches are presented for current and upcoming implementations.

## 2. Vat photopolymerization and nanocomposites

VPP is a general term used to describe several AM technologies. It can be defined as the process by which a liquid polymer resin is selectively cured by a light source in a layer-by-layer manner from a 3D model. The VPP processes include stereolithography (SLA), digital light processing (DLP), continuous digital light projection (CDLP) and two-photon polymerization (2PP). For ease and simplicity, we will discuss SLA and DLP, and further in this study, the term VPP will only describe SLA and DLP. SLA was the first commercial 3D-printing method, which was developed by Charles Hull in 1988. Since then, many advancements in this process have emerged including DLP and CDLP. The high resolution, part strength, and ability to make intricate polymer parts of VPP processes are some of the key features that distinguish them from other AM techniques. The key differences in the common VPP processes are listed in Table 1.

**Table 1:** A summary of the different processes in vat photopolymerization. Most data are taken from commercial supplier's websites

Process	Light source	Lateral resolution *x*–*y* res [μm]	Layer thickness *z* res [μm]	Printing speed [mm h^−1^]	Main features
SLA^a^	Laser beam	6.5–25	25–300	14	Most common VPP technology
					Either be a top-down (light source is above) or bottom-up (light source is below the vat) approach
					Slow but highly accurate
					Better surface finish
					UV light is the common light source
DLP^b^	Projector	33–120	25–150	25–150	Printing speed is faster than SLA due to DMD^d^, but the resolution and accuracy is not as high
					DMD is used to rotate and reflect the light
					The image of each layer comprised of rectangular voxels is called the voxel effect
					Bottom-up approach
CDLP^c^	Projector	50–100	0.4–100	500–1000	A new technique introduced in 2015
					Up to 100 times faster than any AM method
					Bottom-up approach
					Vat contains an oxygen-permeable window
					A dead zone is created between the window and the surface of the part being cured, which inhibits the photopolymerization
					Thus, the part being cured is continuously drawn out of the resin vat
					Smooth parts with good surface finish are built

a Stereolithography.

b Digital light processing.

c Continuous digital light projection.

d Digital micromirror device.

A composite is formed by joining or mixing a reinforcement inside a matrix. Polymer nanocomposites are formed when the reinforcement is a nanomaterial and the matrix is a polymer phase. Nanomaterials can be defined as materials that have at least one internal or external dimension in the range of 1–100 nm.^37^ The properties of nanomaterials are different from their bulk counterpart such as lower melting point, high specific surface area, high mechanical strength, and specific optical properties. By approaching the nm scale, there is a significant effect on the surface area to volume (SA/V) ratio. This increase in the SA/V ratio of nanomaterials has an important effect on their geometrical integrity, having the ability to fine-tune different properties compared to the same material in bulk form.^38^ Furthermore, to make use of these unique properties of nanomaterials, they are mixed in polymer resins to enhance the specific desired properties by printing customized 3D objects. The nanocomposites printed by VPP are mostly applied in the health care field, such as drug delivery, tissue engineering, customized bone, and dental implants.^10,13,39–46^ Similarly, their properties such as mechanical, electrical, optical, and magnetic properties are also widely studied.^47–59^

## 3. Configuration, classification and factors affecting vat photopolymerization

### 3.1 Configuration of stereolithography apparatus

**Fig. 1:**
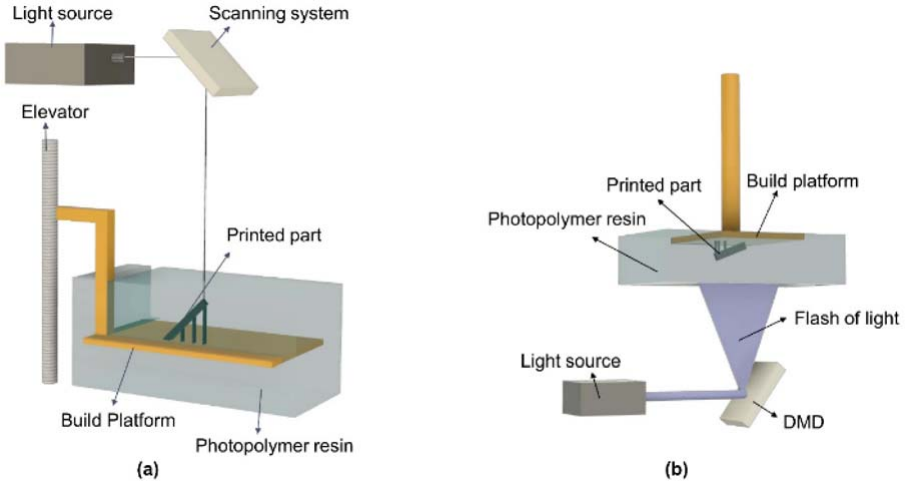
Graphical representation of (a) top down (vector scan) and (b) bottom up (mask projection) approach.

The typical VPP apparatus is shown in Fig. 1(a) and consists of five main parts including a resin vat, recoater blade, build platform, light source, and control systems. The liquid photopolymer resin is placed in the vat. The recoater is used to distribute and swipe the liquid resin when a layer is cured. The build platform attached to an elevator moves upside down and the part is built on it. The light source, usually a laser light of different types (diode, He–Cd, and argon), hits the resin surface from the top. Finally, the control system controls the overall print process, which contains process, beam, and environment controllers.^6^

### 3.2 Classification of printing processes

The printing process in VPP can be classified based on the platform movement and type of light exposure. In this case, the main theme is the same for all, *i.e.*, building a 3D object layer by layer from a CAD model upon exposure to light. However, there are some key differences among them, which are described below.

#### 3.2.1 Platform movement

The movement of the build platform in the *z*-axis defines the printing process. The two different system configurations based on platform movement are the top-down and bottom-up approaches. The top-down approach is shown in Fig. 1(a), where the build platform lies initially in the resin just above the surface. The build platform depth in the resin describes the layer thickness. Upon exposure to a light source, the first layer is cured on the build platform. Then, the build platform is lowered down in the resin at the same height as defined by the layer thickness. The recoater blade swipes the resin and a fresh layer is now exposed to the light. This process continues until a 3D final part is built.

The second configuration is the bottom-up approach, as shown in Fig. 1(b). In this approach, the build platform lies initially at the bottom of a shallow resin tank, which is known as the vat. The bottom of the vat is transparent and the light source hits the resin through this transparent window. Upon exposure to a light source, the first layer is cured, the build platform moves upwards, and a fresh resin comes between the vat window and the already cured layer. This new fresh layer of resin is now exposed to light and the process continues until the part is finished. However, there are some drawbacks related to the top-down approach. Specifically, gravity deteriorates the resin surface, and therefore the layer thickness is difficult to control in this setup. Also, the equilibrium of the photopolymer resin is disturbed as the build platform moves downwards in the resin. Therefore, the recovery of the equilibrium state consumes some extra time, which affects the whole print process given that no printing occurs at that moment. Furthermore, oxygen is always in contact with the fresh resin layer, which inhibits photopolymerization, thus resulting in incomplete curing. Moreover, the sufficient resin is required in this approach as the whole part is built inside the resin. Also, surface tension and viscosity can create a problem when swiping the resin around the part being build.

Some of the problems encountered in the top-down approach can be alleviated by employing the bottom-up approach. The part height is independent of the vat depth, as the part is built facing downwards and built outside the resin, and thus objects with large heights can be printed with less material, resulting in no or little material wastage. Given that the layer thickness is controlled by the platform movement and not by the resin parameters, a smaller layer thickness can be achieved precisely. Also, this approach offers better surface quality and vertical resolution than the top-down approach.

#### 3.2.2 Light exposure

Normally two types of light exposure strategies are applied in VPP, which are the vector scan and mask projection approaches. In the vector scan (point-wise) approach, a UV light selectively cures the resin point wise, as shown in Fig. 1(a). The traditional stereolithography commonly refers to this type of setup. The top-down and bottom-up approaches can both use vector scans as light exposure. The commonly used wavelength range of the laser is 355–405 nm, where most photoinitiators have the maximum absorbance.^60^ Alternatively, in the mask projection (layer-wise) approach, the whole layer of resin is cured at once and the same steps are followed as discussed for the bottom-up processes. The difference between these two strategies is the way the light source hits the resin. Here, the UV light remains stationary and cures the whole layer at a time. The light source comes from a projector that uses a digital micromirror device (DMD).^61^

The mask projection method enables a shorter print time but compromises the accuracy. The point-wise approach takes more time but can print a more complex part with intricate details and high resolution. If there are no time constraints, then the point-wise approach is preferable for performing research in academia because of its high accuracy, which may not be obtainable by fast DLP printers. Most of the literature studies used commercially available printers. However, some made their own customized printers,^62–64^ while some modified existing systems in their research.^46,65^

### 3.3 Factors affecting the printing process

VPP-based 3D printing is a complex process and is affected by many parameters. Some factors are technical, which are related to the apparatus, while the others are the resin parameters. The factors related to the apparatus are the layer thickness, laser type, laser power, laser speed, hatch spacing, machine instability, *etc.*

Alternatively, the factors related to the resin are the penetration depth, critical energy, viscosity, *etc.* All these parameters are important and have a substantial effect on obtaining the desired properties of the final part.

The curing precision determines the printing details and is related to the cure depth, *C*_d_, and cure width, *C*_w_. The cure width at the surface of the resin is the greatest because of the parabolic shape formed by the laser hitting the resin. In modelling the photocuring behaviour in stereolithography, two assumptions are made, *i.e.*, the laser beam hitting the resin is Gaussian and the photopolymer resin obeys the Beer–Lambert law. An important theoretical relationship can be observed based on these assumptions, as shown below. The derivation and more technical details can be found in ref. 66.*C*_d_ = *D*_p_ ln(*E*_max_/*E*_c_)where *C*_d_ is the cure depth of the resin, *D*_p_ is the penetration depth of the laser in the resin at which the light intensity is reduced to 1/*e*^2^ of the resin surface value, *E*_max_ is the peak exposure of the laser hitting the resin, and *E*_c_ is the critical exposure at which solidification starts.

**Fig. 2:**
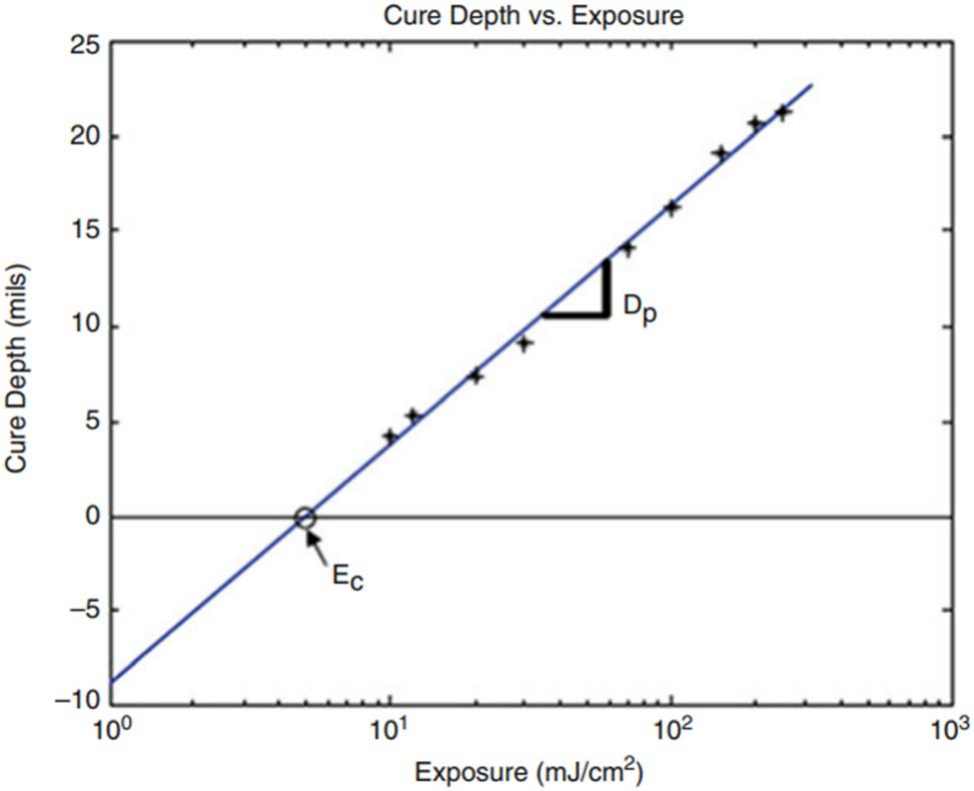
Resin working curve of cure depth *vs.* exposure (reproduced with permission from ref. 67 Copyright, 2015, Springer).

The photocuring behaviour is characterized by a straight line, which is known as the working curve by plotting the cure depth *versus* exposure, as shown in Fig. 2.^67^ The working curve gives information about two key resin constants, *i.e.*, *E*_c_ and *D*_p_. The slope of the working curve defines *E*_c_ at the laser wavelength, while *D*_p_ is the slope of the line at that wavelength. Both the slope and intercept are independent of the laser parameters such as speed and power because *D*_p_ and *E*_c_ are resin parameters.

One of the parameters that affects the printing process is the layer thickness, which is a key factor in describing the part quality and printing speed. Parts with a thick layer thickness are printed faster but at the expense of accuracy, resolution, and surface smoothness, while parts with a small layer thickness take more time to print but have higher resolution, better surface finish with intricate details and improved geometrical accuracy.

## 4. Feedstock materials

The feedstock material used in the VPP process using a polymer nanocomposite is a mixture of nanoparticles dispersed in a photopolymer resin. The resin serves as the polymer matrix, while the nanoparticles help to enhance the desired properties for some specific applications. Both nano reinforcement and the photopolymer resin are briefly discussed in the following section.

### 4.1 Nano reinforcement

A nano reinforcement, as discussed earlier, is a particle having dimensions less than 100 nm. Among the different nanoparticles, cellulose nanocrystals (CNCs), carbon nanotubes (CNTs), hydroxyapatite (HA) and graphene particles are the most widely used. The resulting enhanced properties include mechanical, electrical, biomedical, magnetic properties, where the tensile strength and Young modulus are the most widely studied and enhanced in the context of mechanical properties.

### 4.2 Photopolymer resin

A photopolymer resin is a polymer matrix that changes its properties when exposed to a light source.^26^ The physical change that occurs is the hardening of the liquid resin due to cross-linking when a light source hits the surface. Photopolymers mainly consist of photoinitiators, monomers, oligomers, and some additives, which are briefly described in the following sections. The typical SLA resin consists of monomers (∼25%), photoinitiators (∼5%), and oligomers (∼70%).^6^ Some other additional components such as pigment dyes, inhibitors, dispersion agents, and plasticizers can also be added in low quantities to optimize the properties of the resin.^68^ More comprehensive details about photopolymer resins can be found in ref. 23, 24, 26, 27, 69 and 70

#### 4.2.1 Photopolymer resin formulation

Monomers and oligomers are the main elements of polymer resins, which get polymerized after being exposed to a light source.^60^ The oligomers (prepolymers) are large-size molecules with a very high degree of functionality and consist of a few monomer units. Oligomers are known as the backbone of the polymer chain and greatly affect the end properties of the printed part such as hardness, adhesion, strength, and surface finish.^24^ Photoinitiators are molecules that decompose into reactive species when exposed to a light source and initiate the photopolymerization process. Briefly, the photoinitiators convert the physical energy of the incident light source into chemical energy.^67,69,71^

Additives improve the performance of photopolymer resins. Some common additives include diluents, sensitizers, pigments, and inhibitors. Diluents are added to the photopolymer resin to reduce the viscosity and proper wetting behaviour during the printing process. Generally, the incorporation of nanofillers in polymer resins results in an increase in viscosity, whereas diluents can help reduce it. Inhibitors control the gel time and penetration depth. Sensitizers are beneficial for the formation of reactive species from photoinitiators.^24^ Pigment dyes give colour, coupling agents enhance the nano-filler bond, while chain transfer agents are used to control and modify the crosslinking.^67,70,72^

#### 4.2.2 Photopolymerization process

Photopolymerization (PP) is the cross-linking of polymer chains when a light source is irradiated on them. Likewise, it can be described as the change in the liquid to a solid state by cross-linking of small molecules into large molecules. PP is a complex process and involves numerous micro and macro reactions. PP is an exothermic process and the photoinitiator acts as a catalyst to start the reaction. Two types of PP reactions occur as a result of the photo-initiation effect. Free radical polymerization occurs in acrylates, while cationic polymerization occurs in epoxy and vinyl ether.^27^ A description of photopolymer chemistry can be found in ref. 67 and 69 and briefly summarized as follows.

**Fig. 3:**
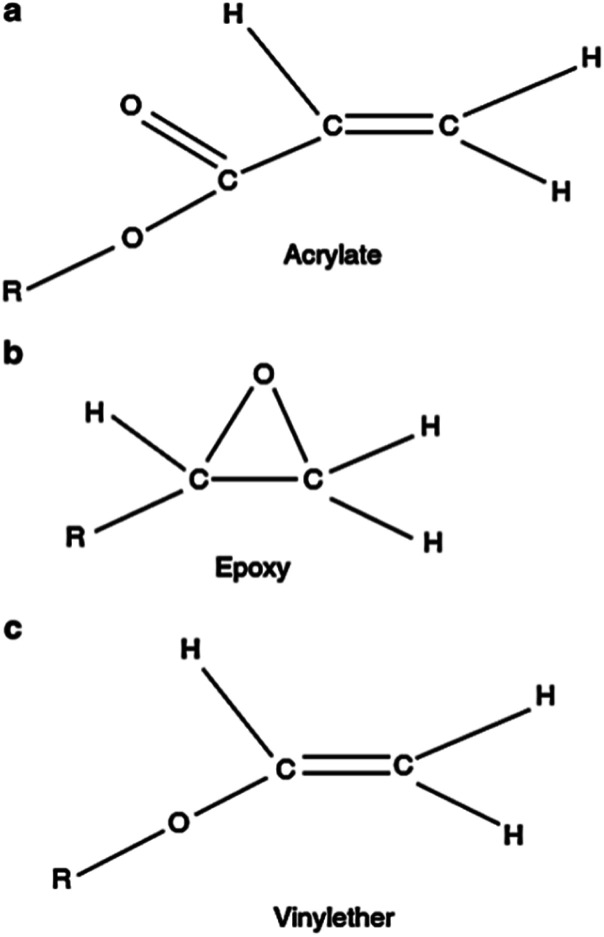
Molecular structure of different monomers. (a) Acrylate, (b) epoxy and (c) vinyl ether (reproduced with permission from ref. 67. Copyright 2015, Springer).

Free radical polymerization occurs in acrylate-based resins and was the first type to be developed commercially. The molecular structure of acrylate is shown in Fig. 3. Once the long chains of polymers are very close to each other, after being initiated by photopolymers, cross-linking in acrylate occurs and solidification starts. The fundamental solidification process of free radical polymerization consists of three steps, *i.e.*, initiation, propagation, and termination.^69,73^

Cationic polymerization occurs in epoxy-based and some vinyl ethers. The epoxy monomer has a ring-type structure, as shown in Fig. 3. Upon reaction, these rings open, forming some sites for other chemical bonds. Because of this ring opening, epoxy-based resins are less susceptible to shrinkage and have less tendency to curl. A photoinitiator catalyst initiates the reaction by generating cations after being exposed to a light source.^69,71^

#### 4.2.3 Challenges in vat photopolymerization-based resins

The early US Patents on acrylate resin were published in the early 90s. Acrylate-printed parts are weak because of their high shrinkage and curling problems. In comparison to acrylate resins, epoxy-based printed parts are harder, more accurate, and stronger with 1–2% shrinkage, which is 5–20% in acrylate resins. However, epoxy-based resins have a different set of problems including brittleness, slow curing speed, and sensitivity to humidity, which can hinder the polymerization process.^24,72^ Thus, to rapidly build parts with less shrinkage and brittleness, the addition of acrylate to epoxy resin is required. In this case, hybrid resins require less exposure time for curing, which is required by both resins independently.^74^ The synergistic approach using two resins can combine the advantages of both, which results in high accuracy and high green strength in the printed part. Currently, some of the commercially available resins are mixed epoxy and acrylates, which can be used and tuned by customers to achieve the desired properties.

## 5. Nanocomposite preparation

### 5.1 Preprocessing

**Fig. 4:**
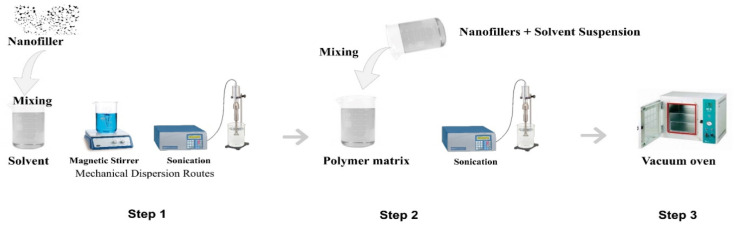
Polymer nanocomposite preparation route.

Different preparation routes have been established to prepare polymer nanocomposite resins for VPP. The most widely used technique is known as solution mixing or solution intercalation, as shown in Fig. 4. The solution mixing method is the most widely used technique for the preparation of polymer nanocomposites, and hence many researchers employ this route. Typically, three steps are involved in this method, as follows: (1) mixing nanoparticles in a suitable solvent by applying different mechanical dispersing routes such as magnetic stirring, high-pressure homogenizing, and sonication or combination of any two. (2) Mixing the nanoparticle suspension in a polymer matrix by sonication. (3) Removal of the solvent by evaporating it in a vacuum oven or blast furnace.^75^

A magnetic stirrer, high-pressure homogenizer, shear mixer, magnetic stirrer, and ultrasonic sonicator (probe and bath) are all used for mixing nanoparticles in the matrix, depending on the size and shape of the nanofiller, the nature of the solvent and the polymer matrix. The magnetic stirrer is a low-cost, simple lab-scale equipment, where a magnetic rod rotates at a high speed to mix the nanoparticles. However, the mixing efficiency of a magnetic stirrer is not very high and it can cause some bubbles or voids to be formed in the nanocomposite, which directly affects the achievable resolution and printability. Thus, to overcome this problem, a vacuum can be used to help remove the air bubbles from the suspension. Ultrasonication is the most efficient way to disperse nanoparticles in the resin. Two types of lab-scale sonicators are commonly available, which are ultrasonic bath and ultrasonic probes. Both types used acoustic sound waves to agitate the nanoparticles in a liquid. Similarly, a high-pressure homogenizer is also used. There is no specific rule to use one standard method for mixing. Basically, the purpose of all methods is the same, *i.e.*, mix the nanoparticles in the polymer matrix. The main goal is to achieve a nanocomposite resin with a uniform dispersion with the nanoparticles homogenously mixed in the polymer matrix. To achieve a uniform and more homogenous mixture, two methods can be used synergistically. Manual mixing or magnetic stirring can be followed by sonication routes or the use of homogenizers and *vice versa*.

### 5.2 Challenges in nanocomposite preparation

The incorporation of nanoparticles in a polymer matrix sounds promising and has the ability to enhance many properties but due to their unique features, there are some challenges associated with the preparation of polymer matrix nanocomposites, as follows.

#### 5.2.1 Agglomeration/aggregation of nanoparticles

Nanoparticles have a high surface area to volume ratio and are held together by van der Waals forces.^38^ Nanoparticles tend to agglomerate easily in the polymer matrix, which significantly affects the final print properties. The problems of nanoparticle agglomeration and the formation of a uniform dispersion are ubiquitous and remain challenging.

#### 5.2.2 Increase in viscosity

Viscosity is an important factor in defining VPP processes, especially in the preparation of polymer nanocomposites as it describes the printability, which should be in the range of (0.1–10 Pa s).^76,77^ The incorporation of nanoparticles in a polymer matrix results in an increase in viscosity due to the aggregation and agglomeration of the nanoparticles. This aggregation occurs as a result of the van der Waals forces that hold the nanoparticles together. Thus, mechanical dispersing routes are applied to break these clusters. In this case, the increase in viscosity due to improper mixing is the main problem in preparing nanocomposites, however less importance is given in the literature to both mixing techniques and viscosity. Sometimes, there is a difference in the results of two different studies that used same the amount of nanofiller and polymer matrix.^78,79^ These biased results support this statement. Specifically, improper mixing leads to the agglomeration of the nanoparticles, which leads to an increase in viscosity.

To reduce the viscosity, diluents are mixed with the polymer.^77,80^ Similarly, an increase in temperature can also help reduce the viscosity but is limited to the resins that are insensitive to heat.^27^ If the viscosity of the nanocomposite resin is too high, printing will take more time, and due to uneven filling, it is more evident that some defects will appear on the printed part. Therefore, proper and optimized shear mixing or sonication will help control the viscosity by breaking down the clusters of aggregates.

#### 5.2.3 Removal of solvent

A solvent is necessary in most nanocomposite preparation routes; however, incomplete removal of the solvent from the resin is somehow challenging. If there are some traces of solvent molecules left behind, they may cause improper curing, and thus affect the printed parts. Therefore, this preparation route needs some modifications and advancements for the nanoparticles to be uniformly distributed inside the polymer matrix with acceptable viscosity and no solvent left behind. Feng *et al.* used a different route with no solvent involved and the viscosity was in the acceptable range.^81^

#### 5.2.4 Blockage of light by nanoparticles

Most nanoparticles are reactive to the UV light source and can scatter or even block the incoming light source. If the concentration of nanoparticles is too high, this leads to agglomeration, which can block the light. Alternatively, a low concentration causes low viscosity and a high cure rate. Thus, an appropriate amount of nanoparticles is required to avoid this problem.

### 5.3 Post-processing

Post-processing steps are required depending on the geometry of the printed part. Given that VPP processes involve the use of liquid resin, therefore when the printed part is taken from the build platform, it contains some uncured and unexposed resin. Thus, post-processing is required including cleaning, curing, polishing, *etc.* The cleaning process improves the quality by removing the impurities and the unexposed resin stuck to the printed part. This is done by pouring or soaking the printed part in some alcohol for a few minutes, followed by rinsing with the same alcohol. Isopropyl alcohol is preferred for cleaning, although other alcohols can also be used. After cleaning the part with alcohol, the extra support structures need to be cut off, which are necessary to support the already cured part during the printing process.

The uncured resin that remains inside the part is completely cured by curing, which allows further crosslinking of the polymer part.^74^ Curing can be done by placing the part in front of a UV light or curing it thermally by placing it in an oven. Both processes enhance the desired properties.^81^ The mechanical and thermal properties of lignin-coated cellulose nanocrystal (L-CNC) nanocomposites were greatly improved when thermally post-cured at a temperature of 120 °C for 40 min.^82^ Nowadays, different commercial suppliers provide all-in-one UV cure, which can undergo both types of processing.^83,84^ Sometimes an additional step is required after curing to make the part smooth by polishing its surface. This is done using sandpaper with increasing grit in the range of 1500–200. A fine smooth surface is obtained after polishing but this process is very time-consuming and parts with complex and intricate surfaces cannot be polished easily.

## 6. Applications

Nanoparticles mixed with polymer matrices can achieve many desired properties and applications. The synergy of nanotechnology and VPP processes provides an efficient combination of applications compared to other AM methods. In this section, we focus on the recent literature from the past three years. Biomedical and mechanical applications are mostly studied, with a focus on electrical, magnetic and thermal properties. Some of the enhanced properties and their use in terms of applications are described below.

### 6.1 Biomedical applications

One of the most promising applications of VPP processes and polymer nanocomposites is their use in the biomedical industry due to the achievable architecture, topography, geometry, wettability, and controlled pore size.^11,13^ The patient-specific medical needs in the healthcare market are increasing daily and are the most important applications of VPP. The health-care field acts as an important driving factor in the development of printing nanocomposites by VPP. Many nano-reinforcement materials are mixed with different types of resins to achieve the desired properties.

3D bioprinting by VPP processes is an advance technique for the fabrication of patient-customized implants and scaffolds for tissue engineering, *etc.* The use of 3D bioprinting to produce customizable artificial tissue matrices with hierarchical structures shows significant potential. SLA-based VPP processes have been widely used to produce tissue engineering scaffolds.^85–91^ Compared to parts printed by FDM, VPP-printed parts provide enhanced fitting, higher resolution, and more comfort to patients.^13,43^

The nanofillers such as hydroxyapatite (HA), chitosan (CH) and zirconia (ZrO_2_) are the most widely studied and their resulting properties in terms of biomedical applications are summarized in Table 2.

**Table 2:** Different polymer nanocomposites produced by VPP processes for biomedical applications

Polymer matrix/Nanofiller	Additives and reinforcements	Highlights	Properties enhanced	Applications	Ref.
Poly(ethylene glycol) diacrylate (PEGDA) + graphene oxide (GO)	GO conc. (0.05, 0.1, 0.25, 0.5, 1 wt%)	Self-prepared printer	Collagen II synthesis increased up to 66%	Human bone marrow scaffolds, cartilage, regenerative medicine	63
	Irgacure 2959 (Photoinitiator PI) 0.5 wt%	Laser diameter 200 μm	GAG secretion increased up to 71%		
		Energy output 25 μJ	Total collagen level increased up to 43%		
		Printing speed 10 mm s^−1^			
Poly(ethylene glycol) diacrylate (PEGDA) + multi-walled carbon nanotubes (MWCNTs)	MWCNTs conc. (0.02, 0.05, 1 wt%)	Printrbot® printer	Maximum Young's modulus achieved was 1 MPa	Neural regeneration	88
		*Z*-Axis resolution 110 μm	Maximum porosity achieved 66%		
		Light wavelength 355 nm	Pulse stimulation 500 μA		
		Energy output 20 μJ			
		Laser diameter 190 ± 50 μm			
		Laser frequency 8–30 kHz			
		Printing speed 15 mm s^−1^			
1,6-Hexanediol diacrylate (HDDA) + hydroxyapatite (HA)	HA conc. (40, 45, 50 vol%)	AutoCera printer	Maximum flexural strength achieved 36.5 MPa	Bone tissue engineering	92
	Diphenyl(2,4,6-trimethyl benzoyl) phosphine oxide (TPO) (PI) 1 wt%	Light wavelength 405 nm	Maximum compression strength achieved 161.9 MPa		
		Light intensity 8000 μW cm^−2^	Maximum porosity achieved 54%		
		Exposure time 8 s	Optimal sintering temp 1250 °C		
		Layer thickness 25 μm			
		Sintering temp 1200–1300 °C			
Acrylate resin + zirconia/hydroxyapatite (ZrO_2_/HA)	ZrO_2/_HA conc. (0, 10, 20, 30 wt%)	Layer thickness 0.02 mm	Maximum compression strength achieved was 52 MPa (HA)	Cancellous bone repair	46
		Sintering temp 1400 °C	Maximum compressive modulus achieved was 4.7 GPa (HA)		
			Maximum compression strength achieved was 39.99 MPa (ZrO_2_)		
WaterShed XC 11122 acrylate + hydroxyapatite (HA)	HA conc. (10, 20, 30, 40, 45 wt%)	LAYING II 1510P printer	Minimum shrinkage in XY and *Z* axis was 36.5% and 13.8%, respectively	Bone scaffolds	89
		Layer thickness 0.02 mm	Volume shrinkage of 65.2%		
		Print speed 20 mm s^−1^	Maximum compressive strength achieved was 12.8 MPa		
		Sintering temp 1200 °C	Proliferation of cells with 500% viability for 48 h		
Polylactic acid–polyurethane (PLA–PUA) + graphene	Graphene conc. (0.5 wt%)	Photon, ANYCUBIC printer	Maximum tensile strength achieved was 68 MPa (62% than casting)	Gyroid scaffold for bone tissue engineering	39
	TEGDMA 37%	Light wavelength 405 nm	Maximum flexural strength achieved was 115 MPa		
	PLA-PUA 62 wt%	Light intensity 20 W	Maximum flexural modulus achieved was 5.8 GPa		
	Irgacure 819 (PI) 1 wt%	*XY* resolution 47 μm			
		*Z* resolution 1.25 μm			
Urethane dimethacrylate (UDMA) + silver-carrying halloysite nanotubes (Ag-HNT)	Ag-HNT conc. (1, 2, 3 wt%)	Light wavelength 405 nm	Maximum flexural strength achieved was 105 MPa (increase in strength up to 25%)	Dentistry	45
	UDMA 60 wt%	Shear strain 0.1 wt%	Maximum flexural modulus achieved was 2.3 GPa		
	Triethylene glycol dimethacrylate (TEGDMA) 40 wt%	Frequency 10 Hz	Relative cell viability of up to 75%		
	(TPO) 1 wt%	Irradiation power 1 mW cm^−2^			
		Layer thickness 0.25 mm			
		Exposure time 12 s			
		*Z* axis resolution 50 μm			
Methacrylate resin + zirconia (ZrO_2_)	ZrO_2_ conc. 78%	No data given	Maximum shrinkage in *Z*-axis was 28%	Dental implants	93
	Dispersant 2 wt%		Optimal sintering temp of 1450 °C		
	PI 5 wt%		Vickers hardness at surface was 1542		
			Vickers hardness at sides was 1268		
			Maximum bending strength achieved was 1268 MPa		
1,6-Hexanediol diacrylate (HDDA) + hydroxyapatite (HA)	HA conc	Light wavelength 405 nm	Maximum compressive strength achieved was 22.5 MPa (CPS scaffolds)	Bone regeneration	94
	TPO	Layer thickness 30 μm	Maximum compressive modulus was 4 GPa (CPS scaffolds)		
		Energy 10 mJ cm^−2^	Maximum porosity achieved was 70%		
		Exposure time 1.5 s			
Formlabs clear methacrylate resin + boron nitride (BN)	BN conc. (0, 0.5, 1 wt%)	Form 1+ printer	Maximum micro hardness was 135 MPa	Bone scaffolds	95
		Light wavelength 405 nm	Maximum compressive strength achieved was 40.2 MPa		
			Maximum compressive modulus was 438 MPa		
			Loss tangent value was 0.14–24		
			Damping constant was 15.4 s^−1^		
Methacrylic anhydride resin + chitosan (CH)	CH conc. (0.5, 1, 2, 4 wt%)	FLASHFORGE printer	Maximum tensile strength achieved was 82 kPa (increase in strength up to 20%)	Tissue engineering, nose architectures	96
	Irgacure 2959 (PI)	Light wavelength 405 nm	Compressive modulus was 910 kPa		
		Irradiation power 15 mW cm^−2^	Relative cell viability of up to 95%		
		Exposure time 15 s			
Methacrylate resin + hydroxyapatite (HA)	HA conc. (20 and 40 wt%)	No data given	Proliferation rate of 400%	Bone repair, tissue engineering	97
	TPO-L (PI)		Swelling was 9% after 28 days		
Photopolymer resin + iron oxide (IO)	IO conc. (1, 3, 5 wt%)	No data given	Relative cell viability of up to 95%	Bone regeneration	98
			Magnetization range was 22.42–66.76 emu g^−1^		
			Maximum compression strength achieved was 70 N m^−2^		
			Young's modulus was 275 N m^−1^		
Poly(ethylene glycol) diacrylate (PEGDA) + hydroxyapatite (HA)	HA conc. (0, 5, 10, 15 vol%)	Anycubic photon printer	Tensile strength was 30 MPa (increase in strength *y* up to 58%)	Repair and reconstruction of load bearing bone defects	99
		Light wavelength 405 nm	Young's modulus 1.9 GPa (increase in modulus by up to 144%)		
		Layer thickness 50 μm	Yield strength of 7%		
		Exposure time 50 s	Maximum toughness achieved was 1.87 MPa		
1,6-Hexanediol diacrylate (HDDA) + ZrO_2_–AlO_2_	ZrO_2_ 80 wt%	CeraBuilder 100 printer	Maximum linear shrinkage was up to 23%	Dental restorations	100
	AlO_2_ 20 wt%	Light wavelength 355 nm	Maximum density achieved was 5.9 g cm^−3^		
	TPO 1 wt%	Layer thickness 0.04 mm	Vickers hardness was 16 GPa		
	Dispersant 2–5 wt%	Scanning speed 2000 mm s^−1^	Fracture toughness was 6.8 MPa m^−1/2^		
			Optimum sintering temperature was 1600 °C		
LithaBone HA 480 E acrylate resin + hydroxyapatite (HA)	—	CeraFab 7500 printer	Intrinsic permeability was 0.75–1.74 × 10^−9^ m^2^	Bone replacement and bone tissue engineering	101
		Light wavelength 460 nm	Compressive strength was 1.60 MPa		
		*X*–*Y* resolution 40 μm	Elastic modulus was 513 MPa		
		Layer thickness 25 μm	Maximum porosity obtained was 80%		
Poly(ethylene glycol) diacrylate (PEGDA) + hydroxyapatite (HA)	HA conc. (40–42 wt%)	*XY* resolution 200 μm	Max compressive strength achieved was 61 MPa (increase in strength by up to 1700%)	Bone regeneration	102
	PI conc. (0.1, 0.25, 0.5, 0.75 wt%)	Layer thickness 30 μm	Elastic modulus was up to 2.3 GPa		
		Laser power 70 mW	Maximum cell viability was 115%		
		Scanning speed 3.9 m s^−1^			
1,6-Hexanediol diacrylate (HDDA) + biphasic calcium phosphate (BCP)	BCP conc. (50.60, 65, 70, 75 wt%)	No data given	Shrinkage at 1300 °C was 22%	Bone tissue engineering	103
	TPO conc. (0.5–2 wt%)		Maximum porosity achieved was 68%		
			Maximum compressive strength achieved was 20 MPa		
			Vickers hardness was 6 GPa		
			Optimal sintering temperature 1300 °C		
Poly(ethylene glycol) diacrylate (PEGDA) + hydroxyapatite (HA)	HA conc. 40 vol%	Home-built printer	Viscosity achieved 200 mPa s	Personalized bone implants	64
		Light wavelength 380–420 nm	Optimal sintering temperature 1300 °C		
		Irradiation power 0.5 mW cm^−2^	Tensile strength was 5 MPa		
		Energy per layer 5–10 mJ cm^−2^			

Zhou *et al.* investigated the effects of graphene oxide (GO)-based gelatin nanocomposite scaffolds on chondrogenic differentiation of human bone marrow mesenchymal stem cells. They successfully produced GO-incorporated a polymer nanocomposite (GelMA–PEGDA–GO) with a hierarchical structure using SLA 3D printing. The resultant scaffolds possessed superior mechanical properties and outstanding biocompatibility. The GelMA–PEGDA–GO scaffolds exhibited a significantly higher protein adsorption capacity than that without GO. In addition, following the GO-induced chondrogenic differentiation of hMSCs, the resulting nanocomposite scaffolds significantly increased the amounts of glycosaminoglycan (GAG) by up to 71%, total protein, and collagen, and thus are a potential option for use in the future of cartilage regenerative medicine applications.^63^

In another study, Se-Jun *et al.* printed scaffolds for nerve regeneration and produced well-dispersed multi-walled carbon nanotube (MWCNT) hydrogel-based nanocomposites with controllable porosity. They showed that customizable MWCNT scaffolds are great candidates for fostering neural differentiation, making them a promising approach for upcoming neural regeneration applications. This study found that scaffolds containing MWCNTs significantly boosted neural stem cell proliferation and early neuronal differentiation compared to the specimens having no MWCNTs. Additionally, biphasic pulse stimulation with a 500 μA current increased neuronal maturity, as measured by quantitative polymerase chain reaction protein expression analyses. The findings of this study showed that electrical stimulation and an electro conductive MWCNT scaffold may be combined to promote neurite outgrowth for therapeutic use in nerve regeneration.^88^

Owning to its high biocompatibility, 3D printing of hydroxyapatite (HA) bioceramic has been widely investigated for application in bone tissue engineering and bone defect healing. Numerous studies have used HA nanoparticles due to their biocompatibility and usefulness in patient-specific needs. Hydroxyapatite is a bioceramic material, making bone contents of up to 65%.^104,105^ HA-reinforced biocompatible composite resin is a perfect replica for making synthetic bone scaffolds compared to natural bone.

Feng *et al.* successfully fabricated HA bioceramic scaffolds by using SLA 3D printing. The major issues associated with HA bioceramic scaffolds, such as dispersion, sintering, mechanical characteristics, and biocompatibility, were examined in their findings. To fabricate HA bioceramic scaffolds, various solid loading slurries were used. Different concentrations of nanofiller (40, 45, and 50 vol%) were used in this study. Increasing the HA concentration resulted in a higher viscous resin, which produced more defects in the printing process, worse dispersion of the nanocomposite slurry, and less homogeneous microstructure. A highly dispersed HA-resin slurry was produced using Solsperse 17000 as the dispersant. The optimum solid loading and dispersant dose were determined to be 2 wt% and 50 vol%, respectively. The sintering of the HA bioceramic scaffold was also investigated. Here, 50 vol% solid loading and 1250 °C were chosen as the optimum values. The study of the mechanical properties and biocompatibility of the HA bioceramic scaffolds showed that these scaffolds have good potential for bone tissue engineering.^92^

Cao *et al.* reported the fabrication of porous scaffolds of zirconia/hydroxyapatite (ZrO_2_/HA) nanocomposites for application in bone repair by using DLP and evaluated their performance in detail. This study demonstrated that by varying the proportion of the two ceramic materials, the scaffolds may reach a specific range of controlled mechanical properties. According to the reported results, the compressive strength of the scaffold improved to 52.25 MPa when HA (10 wt%) was introduced compared to 39.99 MPa for the ZrO_2_ scaffold. However, as the quantity of HA increased to 20 wt%, the compressive strength started to decrease. Also, in the healing phase of bone damage, these nanocomposite scaffolds could maintain good mechanical properties and reduce the effect on the function of the damaged part.^46^

Tissue engineering is an interdisciplinary area that uses engineering applications in the field of biological sciences to find solutions to dysfunctional tissues or damaged bones. Owing to the fact that the printing of biocompatible and biodegradable polymer nanocomposites can be easily achieved using VPP, it is widely used nowadays to print customized scaffolds for tissue engineering applications.^39–41,46,92,96,106^ In this case, the function of a scaffold is to support a defective part and is not a permanent solution.^13^

Zeng *et al.* printed HA scaffolds for personalized tissue substitutes with excellent mechanical and biocompatible properties. The HA concentrations of 10, 20, 30, 40, and 45 wt% were used in this study. It was found that increasing the nanofiller concentration has a direct effect on the viscosity, as reported by ref. 92. The compressive strength of a bioceramic scaffold is an important index to evaluate the performance of scaffolds. After the sintering of the scaffolds, the shrinkage in the *XY* direction was larger than that in the *Z* direction. Overall, the volume shrinkage after sintering was found to be 65.2%. The biological properties of the scaffold were determined by *in vitro* cell culture. According to the cell proliferation results, these nanocomposite scaffolds were found to be biocompatible and suitable for cell growth and proliferation.^89^

**Fig. 5:**
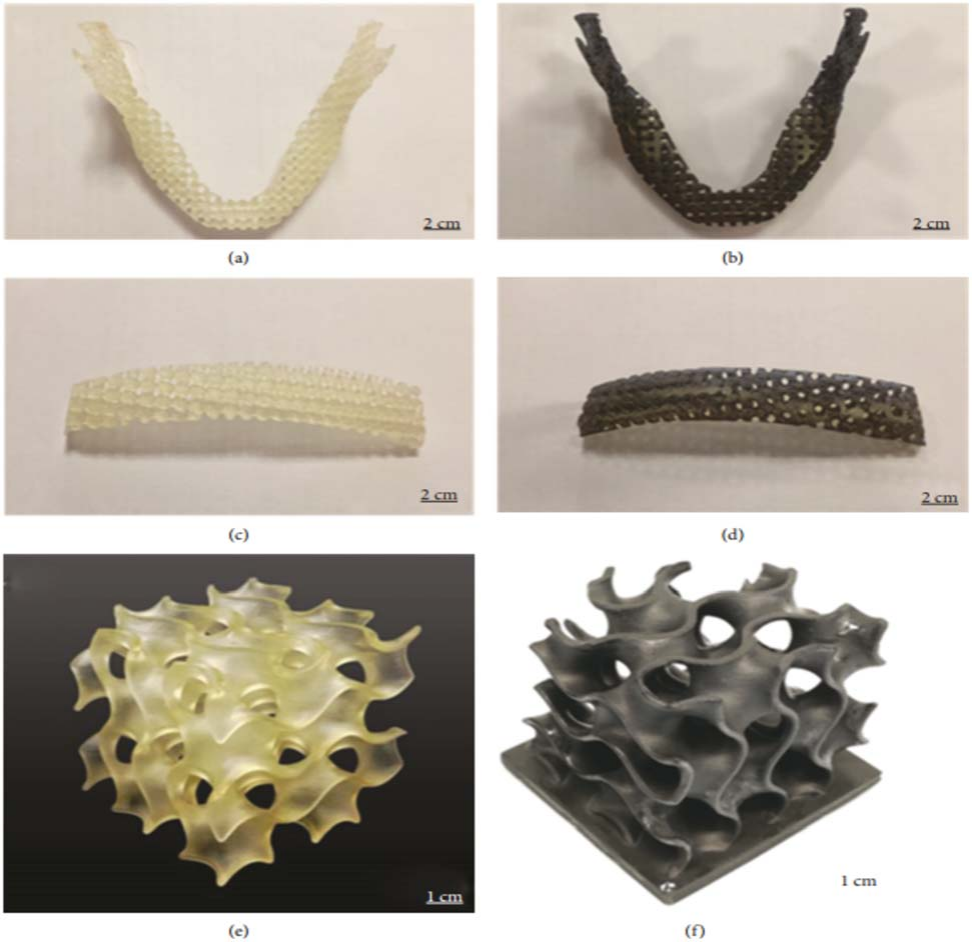
Images of (a) pure UV-cured resin and (b) graphene-reinforced nanocomposite jawbone with a square architecture. Images of (c) pure UV-cured resin and (d) graphene-reinforced nanocomposite sternum with a round architecture. Images of (e) pure UV-cured resin and (f) graphene-reinforced nanocomposite gyroid scaffold for bone tissue engineering (reproduced with permission from ref. 39. Copyright 2018, Hindawi).

Feng *et al.* investigated the bone tissue engineering applications of a graphene-based nanocomposite resin printed by SLA. They used a solvent-free approach for the preparation of a biodegradable nanocomposite resin and studied the mechanical properties of the 3D-printed test specimens with the direct cast method. It was found that the printed specimens had a high tensile strength of 68 MPa, which was almost 62% higher than that of the direct-casted specimens. Similarly, a high flexural strength of 115 MPa and modulus of 5.8 GPa were achieved. The effects of the printing parameters were also investigated, and it was found that a thinner printing layer had high resolution and excellent mechanical properties. Different porous bone structures such as jaw bone, sternum with different architectures and gyroid scaffold for bone tissue engineering were successfully printed, as shown in Fig. 5, which show great potential in biological tissue engineering in comparison to traditional mould-based casting methods.^39^

**Fig. 6:**
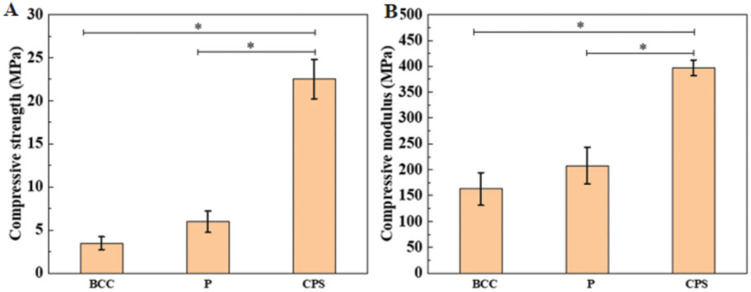
(A) Compressive strength. (B) Compressive modulus. * represents *P* < 0.05 (reproduced with permission from ref. 94. Copyright 2022, WHOICE).

**Fig. 7:**
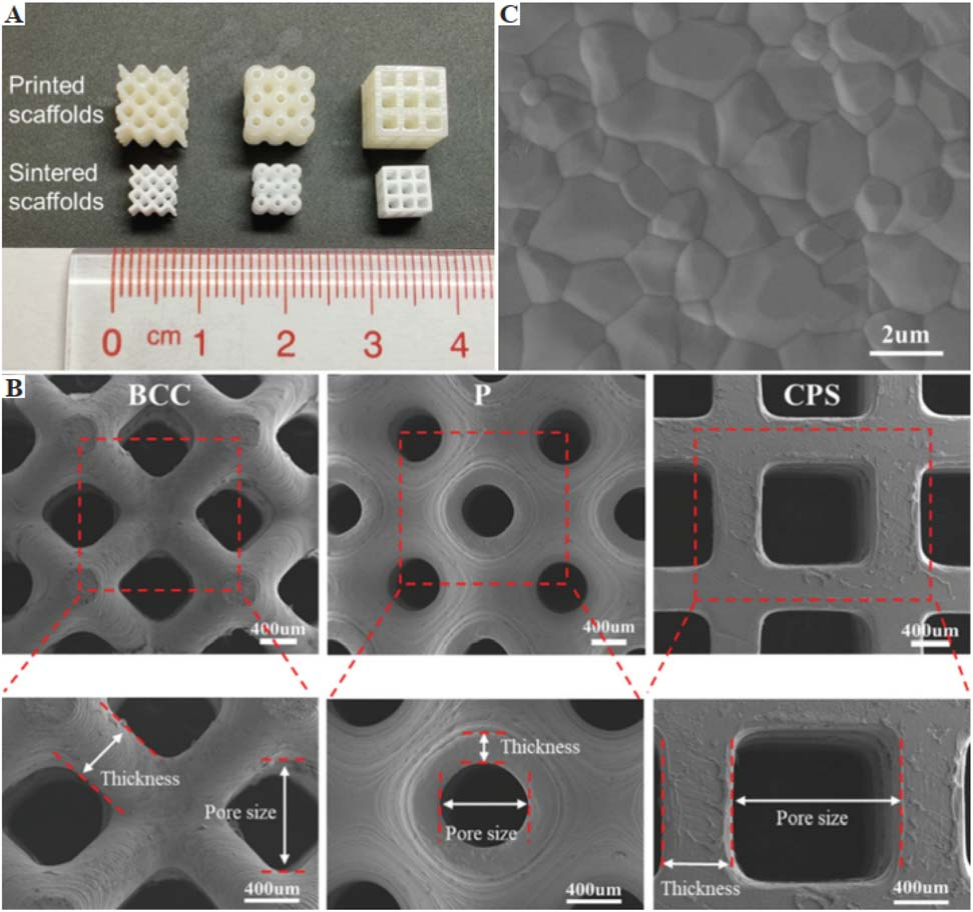
Image of the scaffolds. (A) Printed (upper row) and sintered (lower row) scaffolds. (B) Scanning electron microscopy (SEM) image of surface grains in sintered scaffolds. (C) SEM of the body-centered cubic, primitive, and cubic pore-shaped scaffolds in the top view and their magnified views. Mechanical properties of body-centered cubic, primitive and cubic pore-shaped scaffolds (reproduced with permission from ref. 94. Copyright 2022, WHOICE).

In another study, Liang *et al.* printed hydroxyapatite (HA)-based scaffolds with different porous architectures by DLP 3D printing. The mechanical properties, cell proliferation and morphology of these printed scaffolds were comparatively investigated. In addition, the rheological properties, curing abilities of the nanocomposite resin, debinding, and sintering strategy of the printed part were also systematically investigated. In this study, different types of porous structures such as triply periodic minimal surface-Schwarz primitive (P), body-centered cubic (BCC), and cubic pore-shaped (CPS) nanocomposite scaffolds with ∼70% porosity were printed. According to their results, the CPS scaffolds showed high compressive strength and modulus of up to 22.5 MPa and 400 MPa, respectively, as shown in Fig. 6. Also, the CPS scaffolds performed best in terms of active cell metabolism in comparison to the BCC and P-type scaffolds. These excellent performances of the CPS-based scaffolds were attributed to their small substrate curvature and large pore size, as shown in Fig. 7. This study provided guidance for the selection of different porous scaffolds and printing by DLP.^94^

**Fig. 8:**
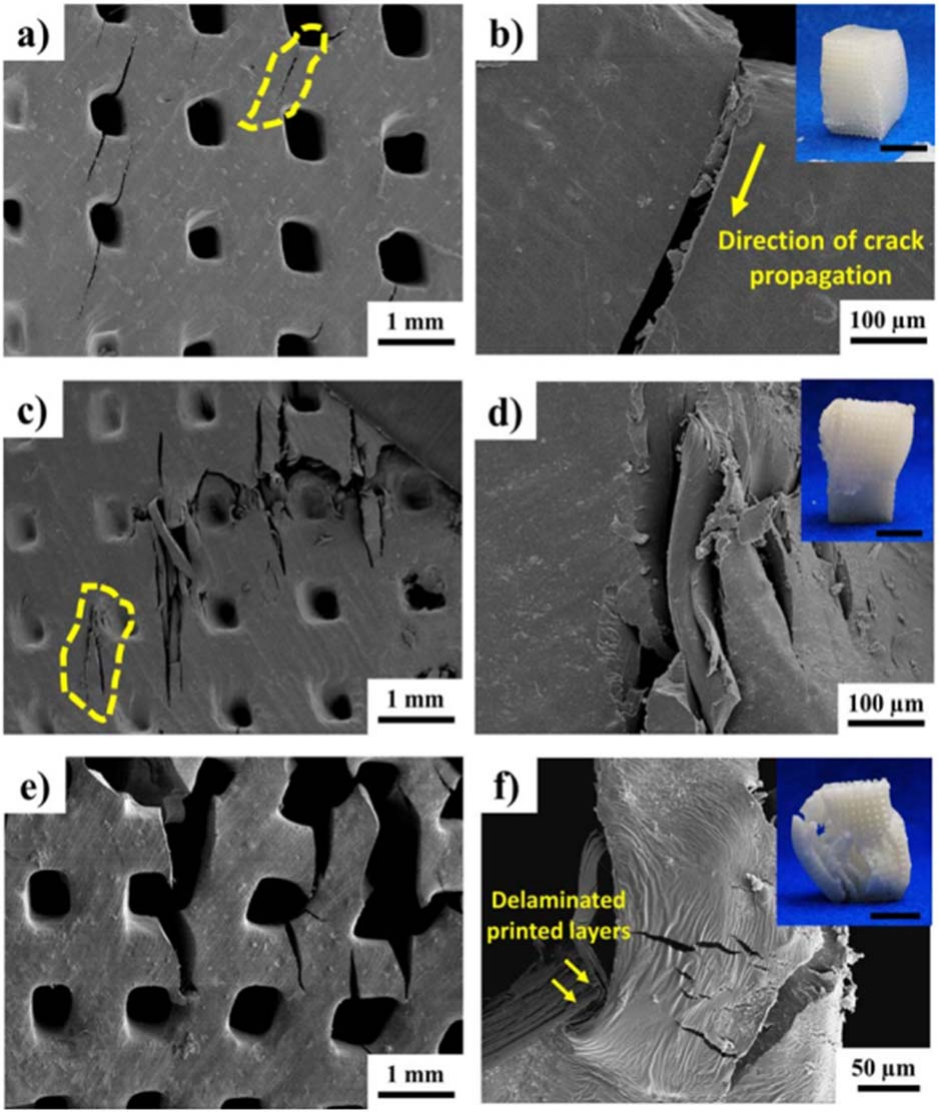
(a) Propagation of oriented (45°) cracks initiated in the walls of larger pores of pure PSP scaffolds. (b) Magnified view of the crack propagation from the pore corner. (c) Brittle fracture of 0.5 wt% boron nitride (BN) scaffolds, (d) sharp cleavage features in the propagation of cracks. (e) Propagation and opening of cracks in 1 wt% BN scaffolds. (f) Buckling and debonding of printed layers due to reduced cross-linking on scaffold structure. Insets show macroscopic images of SLA-printed scaffolds after compressive failure, where all scales are 1 cm (reproduced with permission from ref. 95. Copyright 2017, Wiley Online Library).

Bustillos *et al.* studied boron nitride (BN) nanocomposites and compared the effect of nanofiller addition on the curing of the resin, micro-hardness, damping, and mechanical properties. Different concentrations of BN (0, 0.5, and 1 wt%) were used in this study. Subsequently, it was found that 1 wt% BN filler had a greater effect on the damping response and mechanical properties. It was found that the damping response in the 1 wt% nanocomposite exhibited loss tangent values two folds that of the controlled specimens, as shown in Fig. 8. Similarly, the compressive strength of the 1 wt% nanocomposite was found to be 23.8% more than that of the 0.5 wt% composite. The addition of increased BN concentrations increased the *T*_g_ of the resulting nanocomposites by up to 50 °C. The resulting nanocomposites showed their potential in bone tissue engineering applications.^95^

**Fig. 9:**
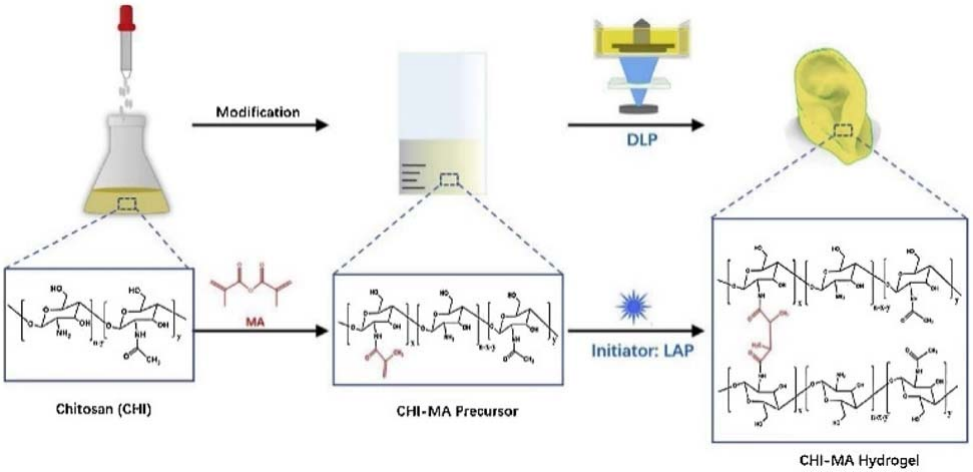
Chitosan-based nanocomposite preparation route. The schematic representation shows the chemical functionalization of chitosan with methacrylate groups and the photo curing process of CHI-MA hydrogels (reproduced with permission from ref. 96. Copyright 2022, Elsevier). For better illustration, visit the online version of this manuscript.

Shen *et al.* proposed the use of a chitosan (CH)-based biocompatible nanocomposite for tissue engineering applications by DLP 3D printing. Different parameters including the concentration of nanofiller, rheology and curing of the nanocomposite resin, and mechanical properties of the 3D printed specimens were investigated in detail. The nanofiller concentration had a direct effect the on mechanical properties, where increasing the CH concentration from 2 to 3 wt% resulted in an increase in compressive modulus from 315 kPa to 910 kPa. According to the cytotoxicity tests, the chitosan-incorporated nanocomposite had excellent biocompatibility. Furthermore, a comparative study was done on the nanocomposite resin with extrusion-based 3D printing and it was found that its compatibility with DLP 3D printing makes this nanocomposite hydrogel an excellent feedstock material with high resolution, and good biocompatibility to print geometric constructions, noses, *etc.*, as shown in Fig. 9.^96^

Guillaume *et al.* studied the bone repair applications of scaffolds printed with hydroxyapatite (HA)-based polymer nanocomposites and investigated their osteopromotive properties in the absence of biologics. Multiple experiments were conducted to check the effects of HA concentration on the osteogenic differentiation of bone marrow stem cells *in vitro* and bone healing kinetics *in vitro*. Different concentrations of HA of 20 and 40 wt% were used in this study. According to their findings, when the nanofiller concentration was 20 wt%, the bone regeneration improved together with osteogenesis and degree of implant osseointegration. In short, this study showed the impacts of biomaterial fabrication *in vitro* and *in vivo* osteogenesis.^97^

Cojocaru *et al.* also studied the bone repair applications of biomimetic scaffolds incorporated with magnetic nanoparticles. Iron oxide (IO) nanofillers were loaded in the polymer resin together with other organic and inorganic fillers. Different mechanical and magnetic tests together with *in vivo* and *in vitro* assessments on the magnetic scaffolds were carried out. Their study revealed that the magnetic susceptibility, mechanical properties and biological fluid retentions were dependent on the final composition of the scaffolds and in the recommended range for bone regeneration applications. The porosity of the scaffolds could be altered by changing the nanofiller concentration in the polymer matrix. The magnetization value of the nanocomposite scaffolds was in the range of 22.42–66.76 emu g^−1^, which is an acceptable value for magnetically loaded materials for application in bone regeneration. Similarly, any printed scaffold should fulfil the requirement of mechanical properties to influence cell functions in tissues. In this case, the compression stress was found to be 70 N m^−2^ and the Young modulus was found to be 275 N m^−2^. The *in vivo* and *in vitro* studies of the nanocomposite scaffolds showed that they are promising feedstock materials for bone repair and regeneration.^98^

In another study, Mondal *et al.* printed acrylated epoxidized soybean oil-based hydroxyapatite (HA) nanocomposites for bone repair applications. Different concentrations of HA nanofillers were used in this study. Increasing the nanofiller concentration increased the strength and toughness of the 3D-printed nanocomposite. When 10 vol% of HA filler concentration was used, the tensile strength, modulus, and fracture toughness increased by 58%, 144% and 42%, respectively, in comparison with the control specimens. The resulting nanocomposite did not show any significant degradation in terms of hydrolytic, enzymatic, or oxidative when incubated for 4 weeks, thus proving its mechanical and chemical stability in the early stages of implantation. Furthermore, the excellent cell viability and proliferation make this nanocomposite efficient for application in bioactivity and bone defect repair.^99^

**Fig. 10:**
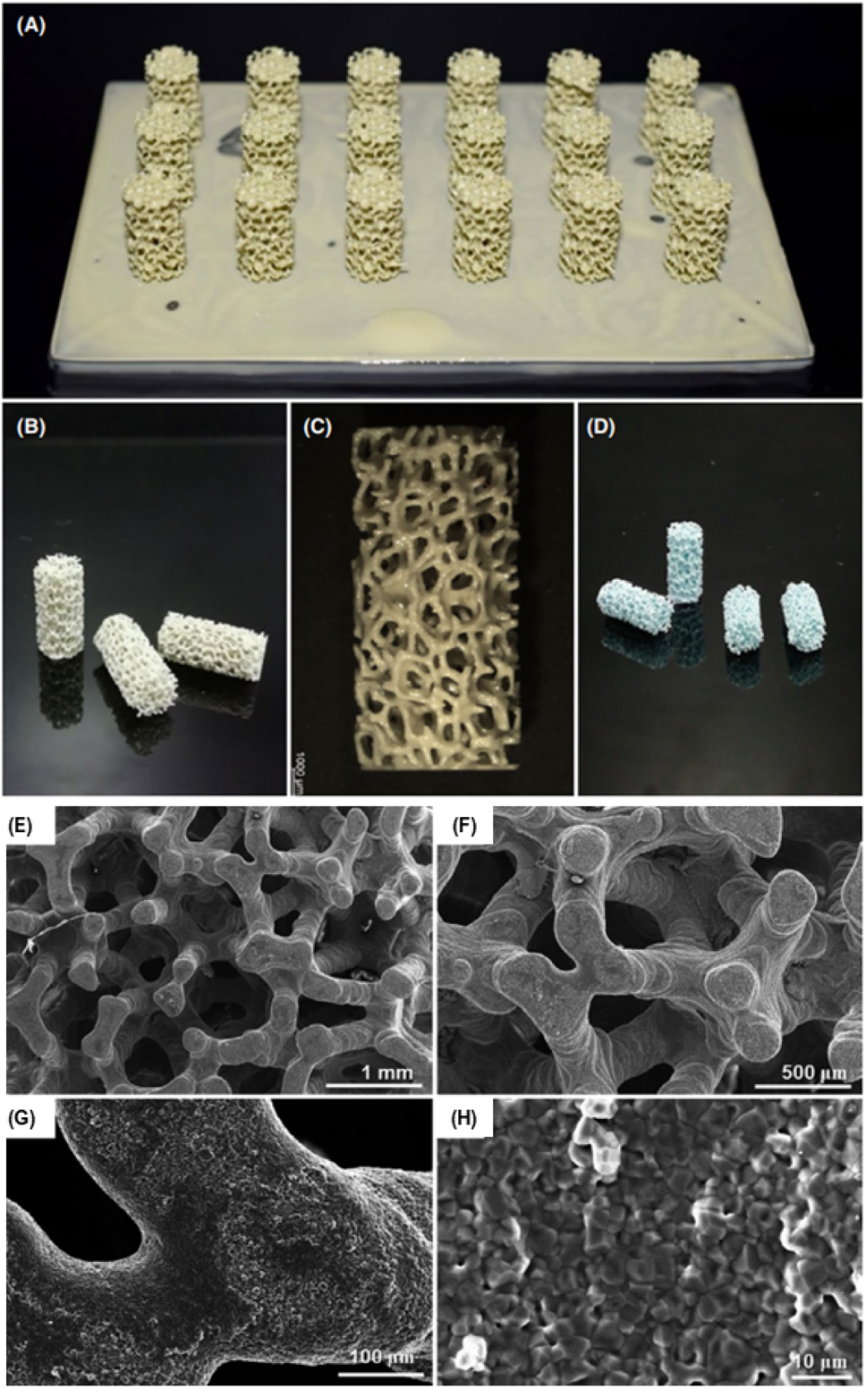
Results of the printing process: green cylindrical samples (A) attached to the building platform just after being printed and (B and C) after being cleaned from the uncured slurry. (D) Sintered nanocomposite scaffolds (E and F) SEM micrographs displaying the 3D trabecular architecture of HA scaffolds, (G) trabecular cross-section and (H) detail of the sintered walls at higher magnification (reproduced with permission from ref. 101. Copyright 2022, MDPI).

Baino *et al.* printed different scaffolds using hydroxyapatite (HA) nanofillers in a photopolymer resin and studied their permeability and mechanical properties. According to their findings, the porosity of the printed scaffolds obtained after sintering was nearly 80%. Similarly, the compression strength and modulus were found to be 1.60 MPa and 513 MPa, respectively, which are in the acceptable range of trabecular bone. These printed nanocomposites showed great potential in the development of porous scaffolds with mass transport properties and bone-like architectures, as shown in Fig. 10.^101^

Achieving optimal conditions in the pre- and post-processing of nanofiller-based bio ceramics is a significant challenge in ceramic-based nanocomposites. Wang *et al.* studied this issue and printed bone tissue engineering scaffolds using a biphasic calcium phosphate (BCP)-based bioceramic slurry. Different parameters and properties such as nanofiller concentration, sintering temperature, hardness, mechanical properties, and *in vitro* performance of the nanocomposites were systematically investigated. Their results showed that when the scaffolds were sintered at 1300 °C, excellent mechanical properties and *in vitro* cytocompatibility were obtained. This study demonstrated porous scaffolds with excellent bioactivity, fidelity and accuracy for bone tissue regeneration.^103^

VPP processes are extensively used in dentistry for the fabrication of orthodontic and prosthetic applications. Normally, when wearing a temporary dental product such as a crown and bridge, saliva is generated by the less tight integration of the dental product and teeth surface. Consequently, bacteria grows in that free space area, causing problems, and eventually the implants need to be removed or replaced, which is costly. Thus, to overcome this problem, antibacterial nanoparticles can be added to the photopolymer resin. Sa *et al.* studied this issue and prepared a dental composite resin with sustainable antibacterial ability using silver carrying halloysite nanotubes (Ag-HNT). Different mechanical, antibacterial and cytotoxicity tests were performed on the resulting nanocomposites. According to their results, the bending strength increased to 25% by using 3 wt% of nanofiller compared to the control specimens. The continuous antibacterial ability of the cured nanocomposite resins was evidenced by culturing *Streptococcus mutans* in the leached resin solution. After 48 h in the leaching solution, the nanocomposite resin showed good cytocompatibility.^45^

Similarly, ZrO_2_ is often used as dental restorative ceramic material due to its excellent mechanical properties, biocompatibility, corrosion resistance, and wear resistance.

Zhao *et al.* investigated a zirconia (ZrO_2_)-based nanocomposite resin for dental implants by DLP 3D printing and studied its fatigue performance simulations. Different parameters were studied to see the effects of heat treatment on the sintering quality of the nanocomposite-based resin. After different testing and microscopy analyses, sintering at 1450 °C for 1.5 h was determined to be the optimum condition. The relative density of the printed nanocomposite was found to be 99.4% and the flexural strength in the horizontal direction was found to be 600 MPa. After the fatigue simulations, it was found that when the pre-angle was smaller, the stress was maximum, and the safety factor was high. The hardness and surface roughness of the nanocomposite-based printed samples were found to meet the requirements of implant abutment.^93^

Chen *et al.* prepared a nanocomposite resin for dental restoration by using ZrO_2_–AlO_2_ nanofillers in a polymer matrix for SLA 3D printing. A fixed loading of 45 vol% nanofiller was used in this study together with 4 wt% dispersant. When the nanocomposite was sintered at 1600 °C, its relative density was found to be 99%, and Vickers hardness was 16.66 GPa. To study the biological properties and biosafety of the nanocomposite resin, rat bone marrow mesenchymal stem cells (rBMSCs) were seeded on its the surface. After their experiments, it was found that rBMSCs have excellent adhesion and proliferation capability on the surface of the nanocomposite. Based on their excellent mechanical properties and biocompatibility, these nanocomposites have broad applications in dental restorations. In short, the biocompatibility of ZrO_2_–AlO_2_ was verified for the first time in SLA-based printed nanocomposites for use in dentistry.^100^

**Fig. 11:**
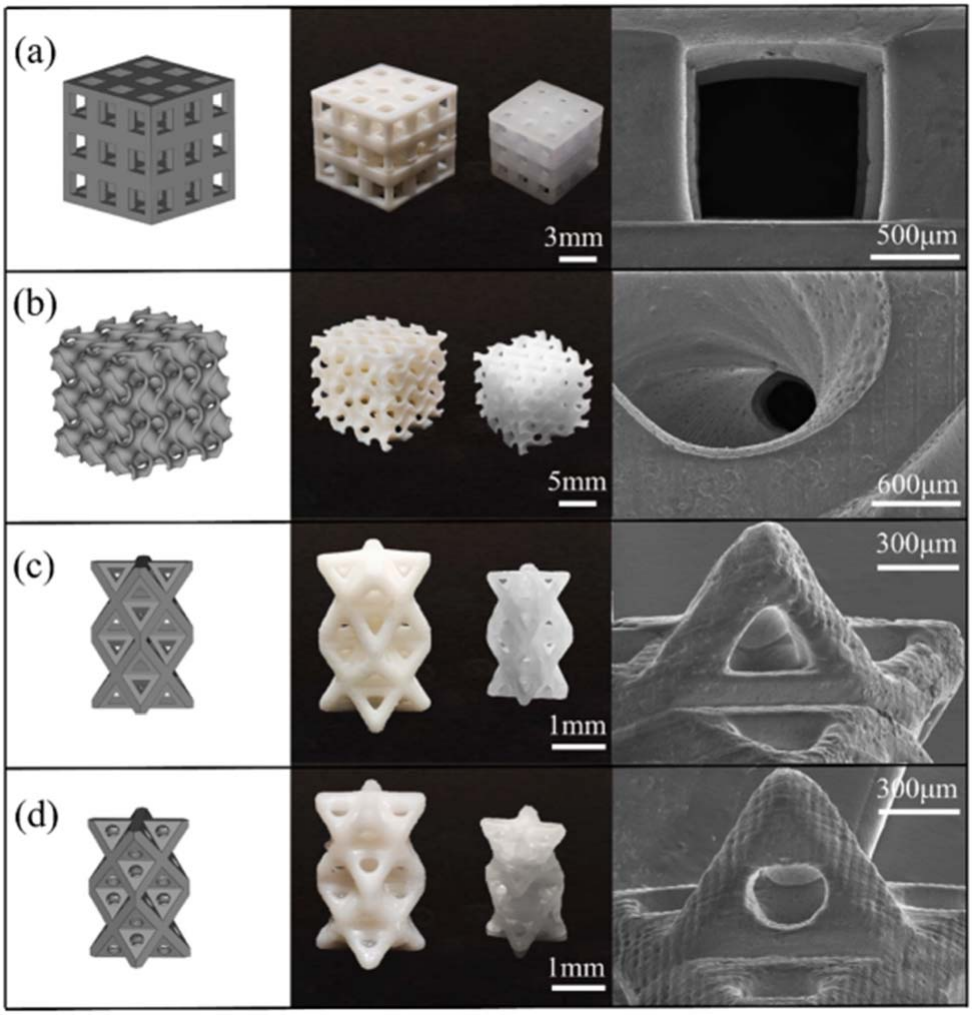
3D models (left column), green bodies, 1300 °C-sintered BCP scaffolds (middle column), and SEM images of various scaffolds (right column): (a) cubic (used for compression test); (b) triply periodic minimal surface (TPMS-G); and (c and d) 3D scaffolds with triangular and circular pores (reproduced with permission from ref. 107. Copyright 2022, Elsevier).

Due to their biocompatibility and excellent mechanical properties, Wang *et al.* used different nanoparticles (GO, titania, sepiolite, and silica) with epoxy-based resin to print dental implants. All the printed parts achieved good dimensional accuracy, as shown in Fig. 11. The nanocomposites printed with sepiolite showed high tensile strength and hardness in comparison to the other printed nanocomposites, which facilitated the fabrication of dental model implants.^107^

AM gained attention in the delivery of customized drugs when the first 3D-printed pill was officially approved by the US Food and Drug Administration (FDA) in 2015.^108^ To date, AM has been used in creating different complex structures. Nowadays, this technology enables the production of customized drug products with appropriate release designs and profiles.^44^ The incorporation of nanomaterials in scaffolds is a feasible route to deliver different drugs to their target.^109^ Abdelrasoul *et al.* mixed gold nanoparticles with a biodegradable polymer polypropylene fumarate (PPF) resin and printed them by the MPExSL technique. Subsequently, the scaffolds were implanted in mice. The results from their experiment proved that gold nanoparticles are promising non-toxic nanocarriers, which can transport genes, drugs, and proteins to deliver them to the target sites.^109^

**Fig. 12:**
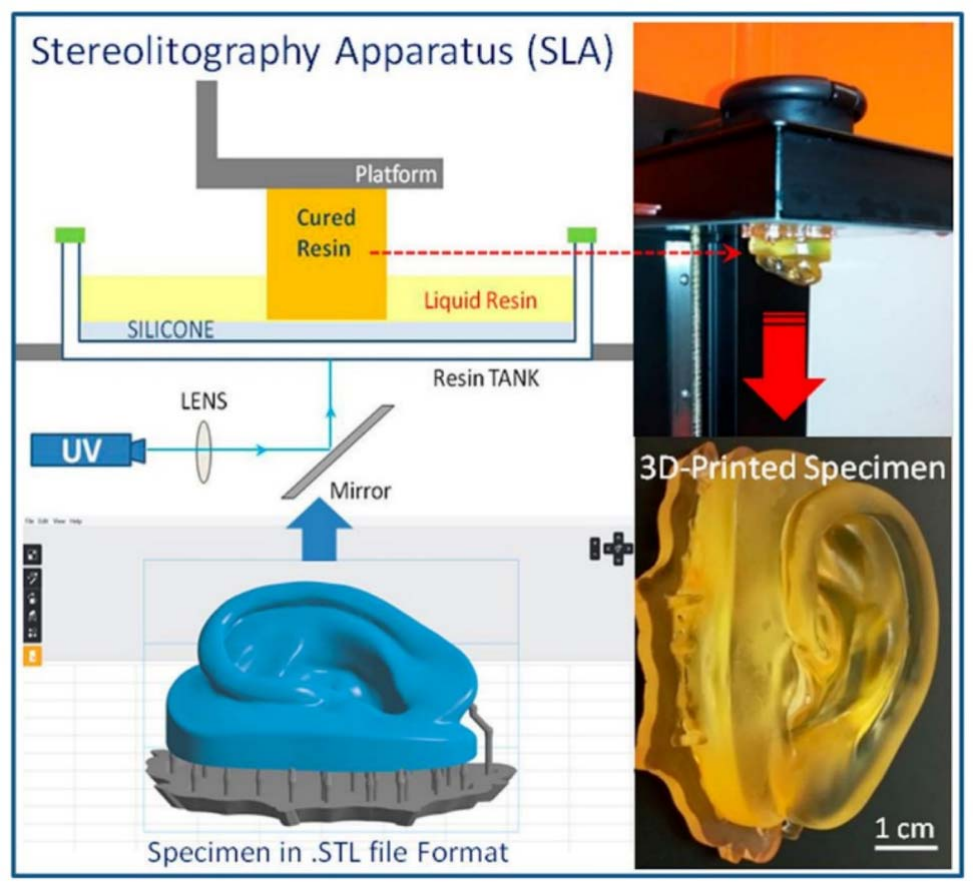
3D printing of human ear construct using PEGDA–CNC hydrogel *via* SLA, which is potentially suitable for tissue engineering applications (reproduced with permission from ref. 111. Copyright 2017, ACS).

Owing to the biocompatibility of CNCs, they are widely used in biomedical applications and have the potential for tissue engineering applications.^43,110^ Palaganas *et al.* mixed CNCs derived from the abaca plant with a polymer matrix (PEGDA). The results showed the improved thermomechanical and physicochemical properties of the nanocomposite resin, which can affect the toughness of the printed part. These enhanced properties show the potential of the formulations for tissue engineering applications. To be specific for the patient's specified needs, a human ear, as shown in Fig. 12, was printed which shows the capability for reconstructive anotia and microtia surgery.^111^

#### 6.1.1 Challenges and solutions

VPP has come a long way in the biomedical industry. However, challenges still exist, especially for biomimetic printed parts. The patient's health and safety are the priority. Thus, due to the strict regulations based on a patient's health, only a few 3D-printed products are available commercially. Recently, the FDA has issued guidance for technical considerations related to AM devices, which is promising for the development of biomaterials in 3D printing. However, the limited material selection hinders the full exploitation of VPP in the biomedical field. Fortunately, the research and innovations in equipment design, material selection, and pre- and post-processing parameters offer propitious opportunities for this technology.

### 6.2 Mechanical applications

Recently, the significant research on polymer materials in VPP makes them a hot topic. 3D-printed polymer nanocomposites become more brittle with an increase in nanofiller content. The main challenge in this case is to retain the ductility, while increasing the mechanical properties. Mechanical properties are the most widely studied properties in the literature. Regarding mechanical properties, the tensile strength and Young's modulus are more studied. The tensile strength is the maximum stress that a material can withstand just before its failure. In the design of new materials and machine parts, the tensile test plays a significant role. Generally, the ASTM D-638 standard is followed to print tensile test specimens for polymer nanocomposites.^112^ The Young's modulus or elastic modulus is one of the most important material properties, which helps to determine the material response to any applied stress. The polymer parts produced by VPP-based 3D printing have poor mechanical properties compared to injection molding, *etc.* In this case, the addition of nanofillers to the photopolymer resin can help improve its mechanical properties. The nanofillers such as graphene oxide, CNC, and CNTs are the most widely studied and their resulting properties in terms of mechanical applications are summarized in Table 3. This section is organized in terms of nanoparticles used such as 0-dimensional, 1-dimensional and 2-dimensional particles.

**Table 3:** Different polymer nanocomposites produced by VPP processes for mechanical applications

Polymer matrix/nanofiller	Additives and reinforcement	Highlights	Properties enhanced	Applications	Ref.
Methacrylate + graphene oxide (GO)	GO conc. (0, 0.05, 0.1, 0.5 wt%)	MakeX printer	Increase in flexural modulus by up to 70% at 0.10 wt% modified GO	Rapid prototyping	59
		Light wavelength 518 nm			
		Exposure time 20 s per layer			
		Layer thickness 60 μm			
		60 micron layer thickness			
Methacrylate + graphene oxide (GO)	GO conc. (0, 0.2, 0.6, 1.0 wt%)	Form 2 printer	Maximum tensile strength achieved was 60 MPa (46% improved)	Drone parts	48
		Light wavelength 405 nm	Maximum compressive strength achieved was 76 MPa	Manufacturing, medical equipment	
		*XY* resolution 25 μm	Compressive modulus was 1.4 GPa		
		Power rating 250 mW	Ductility improved by 66%		
		Build volume 14.5 × 14.5 × 17.5 cm	Increase in toughness by 186%		
Pic 100 resin (EnvisionTEC) + single-layer GO	GO conc. (0.20, 0.5 wt%)	GO *X*- and *Y*-dimensions range 300 nm to 10 μm	Tensile strength achieved was 12.99 MPa (45.3% improvement)	Lattice structures	79
		Thickness range 0.9 ± 0.2 nm	Tensile strength achieved was 14.49 MPa (62.2% improvement) by heat treatment post-curing		
			Strain increased by 12.8%		
Anycubic epoxy resin/TiO_2_	TiO_2_ conc. (0.25, 0.5, 1.0 wt%)	47, 1.25, and 25 l DPI in *x*-, *y*-, and *z*-axes	Maximum tensile strength achieved was 31 MPa	Optical devices	113
		Layer thickness 25–100 μm	Maximum elasticity modulus achieved was 694 MPa (20% higher than the pure polymer)		
			Maximum impact resistance achieved was 12.78 kJ m^−2^		
Acrylate-based “high temperature V2” resin + MWCNTs, GNP, carbon black (CB)	MWCNTs, GNP, CB con. (0.1 wt%)	Light wavelength 395 nm	Relative permittivity increased by 18% for GNP	Piezo-electronics	120
		Layer thickness 60 μm	Relative permittivity decreased by 12% for CB		
			Only MWCNTs increased the dielectric properties		
Tween 20 photopolymer-resin/GNP	GNP con. (0, 1, 2 wt%)	Light wavelength 405 nm	Maximum tensile strength achieved was 35.7 MPa	Solar cells, LEDs, optical devices	123
		Layer thickness 6–8 μm			
		Printer dimensions 1 inch × 14 inch × 22.5 inch			
Flexible polyurethane + polyaniline (PANI) and a graphene sheet (GS)	PANI conc. (1–6 wt%)	IM-96, Carima Printer	Maximum tensile strength achieved was 44.5 MPa (PANI)	Conducting polymers (CPs) with conjugated structures	49
	GS conc. (0.33–2 wt%)		Maximum tensile strength achieved was 69.3 MPa (GS)		
			Sheet resistance improved to 1.19 × 10^6^ ohm sq^−1^		
Spot-HT ABS- photopolymer + CNT	CNTs conc. (0.25 wt%)	Light wavelength 405 nm	Maximum tensile strength achieved was 48 MPa (improved by 70%) post-curing	Functional materials	119
		Cure depth dimension 1 mm × 1 mm	Maximum elongation achieved was 20% (improved by 46%)		
		Layer thickness 6–8 μm	Maximum modulus achieved 885 MPa (post-curing)		
Formlabs grey resin + GO	GO conc. (0.01–0.1 wt%)	Form 2 printer	Maximum tensile strength achieved was 88 MPa (post-curing)	Aerospace, automotive and sports equipment	47
		Layer thickness 15–25 μm	Maximum modulus achieved was 2180 MPa 48.3% (post-curing)		
		Light wavelength 405 nm	Tensile strength was reduced by 17% (solvent addition)		
			Young's modulus decreased by 72.4% (solvent addition)		
Polyurethane + lignin/GNP	Polyurethane acrylate 45–47 wt%	Wanhao 3D printer	Maximum tensile strength achieved was 27.35 MPa (increase in strength by up to 27%)	High strength applications	124
	Morpholine 34–36 wt%	Light wavelength 405 nm	Maximum Young's modulus achieved was 12.68 MPa		
	Tripropylene glycol diacrylate 15–17 wt%		Maximum hardness achieved was 92 MPa (increase in hardness by up to 238%)		
	GNP conc. (0.2, 0.4, 0.6, 0.8, 1.0, 3.0 wt%)				
PIC 100 from Envision TEC + GO	GO conc. (1, 2, 3 wt%)	Light intensity 3900 μW cm^2^	Maximum sheet resistance achieved was 40 × 10^3^ Ω per square	Electronics	125
		Layer thickness 30 μm	Electrical conductivity and mechanical strength were enhanced two-fold with pyrolysis		
		Curing time 7, 11, 18 s			
Polylactic acid polyurethane oligomer (PLA–PUA) + GO	Triethylene glycol dimethacrylate (TEGDMA) 39%	Photon, Anycubic printer	Maximum flexural modulus achieved 3.06 GPa (increase in modulus by up to 14%)	Tissue engineering, personalized medical devices	81
	(Irgacure 819) TPO 3%	Light wavelength 405 nm	Maximum flexural strength achieved was 93 MPa (increase in toughness by up to 28%)		
		*XY* resolution 47 μm			
	GO conc. (0, 0.5 wt%)	*Z* axis resolution 1.25 μm			
		Build volume 115 × 65 × 155 mm^3^			
		Printing speed 0.020 m h^−1^			
Formlabs flexible acrylate resin + GO	GO conc. (0, 0.1, 0.2, 0.3 wt%)	Layer thickness 15–25 μm	Maximum Young's modulus achieved was 6.18 MPa		78
		Light wavelength 405 nm	No significant increase in mechanical or thermal properties due to non-uniform dispersion of GO		
Polylactic acid polyurethane PLA–PUA resin + GO	GO conc. (0.5 wt%)	Photon, Anycubic printer	Maximum tensile strength achieved was 68 MPa (increase in strength by up to 62%)	Porous scaffolds, tissue engineering	39
	(Irgacure 819) TPO 1 wt%	Light wavelength 405 nm *XY* resolution 47 μm	Maximum flexural strength achieved was 115 MPa		
	Triethylene glycol dimethacrylate (TEGDMA, 37 wt%)	*Z* axis resolution 1.25 μm	Maximum flexural modulus achieved was 5.8 GPa		
		UV light intensity 20 W			
		Printing speed 0.020 m h^−1^			
Formlabs grey acrylic resin + GO	GO conc. (0.1, 0.5, 1 wt%)	Form 1+ Formlabs printer	Tensile strength was 23 MPa (50 °C post-curing)	Rapid prototyping	126
		Light wavelength 405 nm	Tensile strength was 50 MPa (100 °C post-curing). Increase in strength by up to 673.6% (for 1 wt% GO)		
		Laser power 120 mW			
		Layer thickness 50 μm			
Formlabs clear acrylic resin + GNP	GNP conc. (0, 0.5, 1, 2, 2.5, 3, 5 wt%)	Form 1+ Formlabs printer	**Before thermal post-curing**	ESD prevention, semiconductors	127
		Light wavelength 405 nm	Tensile strength was 48.8 MPa		
		Laser power 120 mW	Young's modulus was 1.28 GPa		
		Spot size 140 μm	Elongation at break was 35%		
			**After thermal post-curing**		
			Tensile strength was 54.8 MPa		
			Young's modulus was 1.8 GPa		
			Elongation at break was 7%		
			Electrical conductivity was 10^−6^ S cm^−1^		
			Sheet resistance was 10^7^ ohm sq^−1^		
Formlabs standard clear resin + Cu	Cu conc. (0.5, 1, 2 wt%)	Form 2 Formlabs printer	Maximum tensile strength was 77.2 MPa (increased by 33.7% at 1 wt%)	High-end medical applications	117
	Urethane dimethacrylate 55–75%	Light wavelength 405 nm	Maximum Young's modulus was 36.4 MPa (increased 19.4% at 0.5 wt%)		
	Methacrylate monomers 15–25%	Laser power 120 mW	Maximum flexural strength was 109.2 MPa (increased by 33.4% at 1 wt%)		
	Irgacure 819 (TPO) 0.9%	Spot size 150 μm	Maximum flexural modulus was 1.9 GPa (increased by 20.3% at 1.0 wt%)		
			Maximum tensile toughness was 12.4 MJ m^−3^ (increased by 68.5% at 1 wt%)		
			Maximum flexural toughness was 3.2 MJ m^−3^ (increased by 34.2% at 1 wt%)		
			Maximum impact toughness was 11.2 kJ m^−2^ (increased by 30.3% at 1 wt%)		
Formlabs methacrylate resin + lignin-coated cellulose nanocrystals (L-CNC)	L-CNCs conc. (0, 0.1, 0.5, 1 wt%)	Form 1+ Formlabs printer	**Before thermal post-curing**	Electronic components, packages and tissue engineering	82
		Light wavelength 405 nm	Tensile strength was 36 MPa		
		Laser power 120 mW	Young's modulus was 0.67 GPa		
		Spot size 140 μm	Elongation at break was 11%		
			**After thermal post-curing**		
			Tensile strength was 37 MPa (slightly increased)		
			Young's modulus was 0.67 GPa (almost the same)		
			Elongation at break was 11%		
Tetra function polyester acrylate (TPA) + carbon black (CB)	CB conc. (0.5, 1, 1.5, 2 wt%)	Ultrahigh voltage mercury lamp 200 W	Increase in hardness by up to 76%	Visible-light RP machine	118
	1,6-Hexanediol di-acrylate (HDDA) 50%	2000ANSI lumen	Increase in tensile strength by up to 81%		
	TEGO Dispers 680 UV (dispersant) 1.5%	*Z*-axis resolution 0.02 mm	Increase in decomposition temperature by up to 80%		
	IRGACURE 784 (PI)				
Epoxy resin + cellulose nanocrystals (CNCs)	CNCs conc. (0.5, 2, 5, 10, 15 wt%)		15 wt% CNC modulus increased by 100%, glass transition temperature (*T*_g_) increased from 66.5 °C to 75.5 °C and tensile strength increased from 40 MPa to 60 MPa	Reinforcing fillers in structural materials and coatings	128
Acrylate + hydroxyapatite (HA)	1,6-Hexanediol diacrylate (HDDA)	Light wavelength 405 nm	The highest compressive strength achieved was 22.5 MPa	Bone engineering	94
	Diphenyl (2,4,6-trimethylbenzoyl)phosphine oxide (TPO)	Layer thickness 30 μm	The highest compressive modulus achieved was 400 MPa		
		Energy dose 10 mJ cm^−2^	Porosity level was up to 70%		
		Exposure time 1.5 s			
Formlabs clear acrylate resin + graphene oxide (GO)	GO conc. (0.1–2.5 wt%)	Form 1+ Formlabs printer	**Before thermal post-curing**	Biosensing, tissue engineering	122
		Light wavelength 405 nm	Tensile strength was 34 MPa		
		Laser power 120 mW	Young's modulus was 1.5 GPa		
		Spot size 140 μm	Elongation at break was 7%		
		Layer height 100–200 μm	**After thermal post-curing**		
			Tensile strength was 75 MPa (increased up to 100%)		
			Young's modulus was 2.7 GPa (increased up to 90%)		
			Elongation at break was 22%		
Methacrylate resin + cellulose nanocrystals (CNCs)	CNCs conc. (0.5, 1, 5, 10 wt%)	3D systems VIPER printer	Maximum tensile strength achieved was 82 MPa (2.0 wt%)	Rapid prototyping	121
		Light wavelength 355 nm	Maximum young modulus achieved was 4.1 GPa (5 wt%)		
		Laser power 100 mW	Maximum elongation at break was 3.7% (0.5 wt%) with an increase in modulus by up to 57%		
			Maximum flexural modulus achieved was 3.3 GPa		
Poly(ethylene glycol)diacrylate, PEGDA + graphene oxide (GO)	GO conc. (0, 0.3, 0.5 wt%)	HD 2.0 (Robot Factory) printer	Maximum tensile strength achieved was 10.4 MPa (post-cured)	Electronics	129
	Irgacure 819 (PI) 2 wt%	*XY* resolution 50 μm	Maximum compression strength achieved was 11.1 MPa (post-cured)		
		Layer thickness 30 μm	Maximum conductivity achieved was 109.5 × 10^−9^ S cm^−1^		
		Printing time 1.5–2 s per layer			
		Light intensity 10 mW cm^−2^			
Methacrylic (MA) resin (FLGPCL04) + cellulose nanocrystals (CNCs)	CNCs conc. (0.1, 0.5, 1 wt%)	AUTOCERA printer	Maximum tensile strength achieved was 18.8 MPa (increase in strength by up to 43.5%)	Dental model, tissue engineering	130
		Light wavelength 405 nm	Maximum Young's modulus achieved was 240 MPa		
		Laser intensity 7500 μW cm^−2^	Maximum elongation at break was 34%		
		Exposure time 10–30 s			
		Laser power 100 mW			
Bisphenol-A epoxy diacrylate (E-44) + graphene oxide (GO)	GO conc. (0.01–0.12 wt%)	Hygieo WIZART printer	Maximum tensile strength achieved was 78.3 MPa	Prototypes *etc.*	131
	Irgacure 250 1.5 wt%		Maximum Young's modulus achieved was 2.64 GPa		
	PAS 33 (sensitizer) 0.75 wt%		Maximum elongation at break was 7.6%		
			Maximum bending strength achieved was 87.3 MPa		
			Maximum flexural modulus achieved was 2.02 GPa		
			Maximum impact strength achieved was 77.4 J cm^−1^		
			Minimum volume shrinkage was 0.12%		

#### 6.2.1 0-Dimensional particles

0-Dimensional nanoparticles have a length, width, and height of less than 100 nm. Titanium dioxide (TiO_2_) is a 0-dimensional nanoparticle and is most widely studied in this category. Aktitiz *et al.* used TiO_2_ nanoparticles in epoxy photosensitive resin at various concentrations (pure, 0.25, 0.5, and 10 wt%) and studied their effect on different strengths. The maximum tensile strength achieved was found to be 31 MPa, which was lower than that of the control specimen. Similarly, an increase in concentration reduced the impact strength, while enhancing the modulus of elasticity. Because of the hydrodynamic impact of the inclusion of the stiff nanoparticles, the modulus of elasticity was enhanced by 20% compared to the control specimens. The tensile and impact tests demonstrated that the nanocomposite brittleness level increased with an increase in nanofiller concentration. At 337 °C, all the polymer constructions exhibited a 5% weight loss, but no significant changes in their thermal stabilities were seen since the reinforcement was placed at 1% and below.^113^

Guo *et al.* produced fine lattice structures using TiO_2_ nanoparticles in a homemade DLP 3D printer. By optimizing the sintering and debinding, different structures of porosity of up to 80% were obtained. The compressive strength of the structures was affected with a decrease in their porosity. Alternatively, the compressive strength increased from 1.13 MPa to 1.50 MPa with an increase in porosity. By comparing the as-sintered and before-sintered samples, it was found that the *X*–*Y*–*Z* axis shrinkage direction of as sintered structures was anisotropic. These printed nanocomposites showed potential applications in bone tissue engineering, filters, and radiators.^114^

In another study, Mubarak *et al.* proposed a novel approach to improve the mechanical and thermal properties of TiO_2_ nanocomposites by SLA 3D printing. Different crystalline phases of TiO_2_ (anatase and rutile) were used in this study. The annealed anatase printed nanocomposites had a low energy bandgap, high strength of 47 MPa, and modulus of 2.2 GPa. The strength and modulus of the printed nanocomposites improved by 103% and 32% compared to the control specimens, respectively. Due to the low energy bandgap, a good dispersion of nanofiller in the photopolymer matrix was obtained.^115^

Billings *et al.* investigated TiO_2_-based nanocomposites for public health applications. A fixed content of 1 wt% of nanofiller was used throughout to avoid an increase in viscosity. A tensile strength of 29 MPa was obtained in this study. The resulting nanocomposite was clear and transparent and the dispersion of the nanofiller was partially evidenced.^116^

**Fig. 13:**
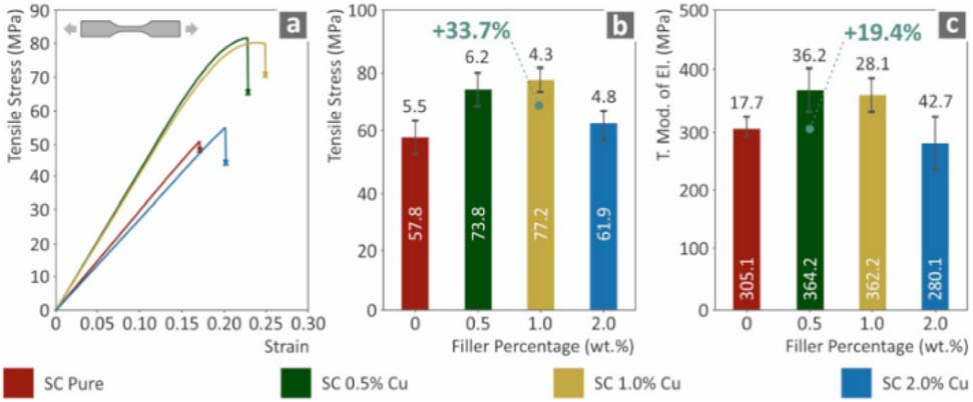
Typical mechanical properties of the control, 0.5, 1, and 2 wt% specimens. (a) Tensile stress. (b) Tensile stress at break. (c) Tensile elastic modulus (reproduced with permission from ref. 117. Copyright 2022, MDPI).

In another study, Vidakis *et al.* investigated the biocidal performance of copper-enhanced polymer nanocomposites with excellent mechanical properties. The agar-well diffusion technique was used to assess the antibacterial activity of the nanocomposites. Compared to the control specimens, the results of the tensile tests for the nanocomposites filled with 1.0 wt% ratios showed an improved mechanical performance of about 33.7%, as shown in Fig. 13. This concentration also demonstrated a sufficient antibacterial performance compared to lower filler loadings, making it an efficient nanocomposite for use in medical applications using the SLA process.^117^

Chiu *et al.* investigated the mechanical and thermal properties of a carbon black (CB)-incorporated photopolymer nanocomposite. Their findings demonstrated that the prototype made from pure photopolymer tended to have poor mechanical and thermal stability. However, with the addition of different concentrations of CB and dispersant in a suitable composition, the resulting nanocomposite exhibited a reduced curing time, enhanced mechanical properties, and thermal and dimensional stability. The resulting nanocomposite showed excellent thermal stability with an increase in its decomposition temperature by up to 80%, enhanced hardness of up to 76.6% and tensile strength of up to 81%.^118^

The mechanical and biological properties of scaffolds are significantly influenced by their morphologies and structures. Liang *et al.* printed nano-hydroxyapatite (HA) bone scaffolds with multiple porous structures by DLP 3D printing. Different properties such as rheological, curing abilities, debinding, and sintering strategy were systematically investigated. The compressive qualities and *in vitro* cell assessments of the scaffolds, including cell growth and attachment morphology, were comprehensively evaluated. According to their findings, the scaffolds had a high modulus (400 MPa) and compressive strength (22.5 MPa). Additionally, the CPS scaffolds had the most active cell metabolisms compared to the other two structures, which may explain the smaller curvature and bigger pore size of the substrate.^94^

#### 6.2.2 1-Dimensional nanoparticles/wires

1-Dimensional wires have one of their length, width or height in the nm scale, *i.e.*, less than 100 nm. In terms of 1-dimensional nanofillers, carbon nanotubes (CNTs), multi-walled carbon nanotubes (MWCNTs), and cellulose nanocrystals (CNCs) are widely used.

CNTs are desirable nanofillers because of their high strength and Young's modulus. Polymer parts produced by VPP methods have poor mechanical properties compared to injection molding, *etc.* In this case, the addition of nanofillers to the photopolymer resin for SLA can help improve their mechanical properties. Eng *et al.* developed a CNT-based photopolymer resin for SLA 3D printing. Nanofillers agglomerate easily in the polymer resin; however, if the nanofiller is well dispersed, it will not agglomerate. Here, examining appropriate dispersion mechanisms and processes is necessary to achieve a good dispersion, which is essential for enhancing the mechanical properties. A fixed concentration of CNTs (0.25 wt%) was used throughout the study. Similarly, thermal post-curing significantly improved the mechanical properties of the printed parts and a maximum tensile strength and modulus of 46 MPa and 885 MPa were achieved, respectively. Overall, the tensile stress and elongation of the 3D-printed components were enhanced by 70% and 46%, respectively, compared to the control specimens. The acquired tensile strength was somehow equivalent to the components made using traditional techniques because of the substantial improvement. The addition of CNTs to photopolymers can result in additional desirable qualities other than mechanical properties, such as better thermal and electrical conductivity and a slower rate of deterioration due to the UV-absorbent nature of CNTs.^119^

Mitkus *et al.* investigated the post-curing, microstructure, viscosity, cure depth, and dielectric properties of different nanofiller-based composites such as MWCNT, GNP and carbon black (CB). This study revealed how nanoparticle dispersions affect the different properties of printed nanocomposites. Different suspensions of nanofillers were created and a reduction in velocity was observed. Similarly, the addition of 0.1 wt% nanofillers resulted in a poor dispersion, leading to a cure depth of 90% compared to the control specimens. The graphene nanoplatelets (GNP) composite had the biggest increase in relative permittivity by up to 18% (at 1 kHz). However, carbon black (CB) resulted in a 12% reduction in relative permittivity (at 1 kHz). Besides MWCNT composites, where the dissipation factor decreases with frequency, no variations in the dissipation factor were observed. For further research in this area, it was suggested that MWCNTs and GNPs be used in combination with ethanol as a dispersant to improve their dispersion. Similarly, post-curing of the resulting nanocomposites affects some of their properties. Subsequent post-curing showed a slight decrease in relative permittivity and a dramatic decrease in dissipation factor with an increase in post-curing time.^120^

Cellulose nanocrystals (CNCs) were used in a photopolymer matrix for the first time by Kumar *et al.*, using SLA 3D printing. In their study, different concentrations (0, 0.5, 2, and 5 wt%) were used in the polymer matrix. Increasing the nanofiller concentration up to 5 wt% resulted in an increase in mechanical properties, which subsequently decreased. These excellent mechanical properties were due to the high level of dispersion and the intimate contact of the nanofiller with the polymer matrix.^121^

**Fig. 14:**
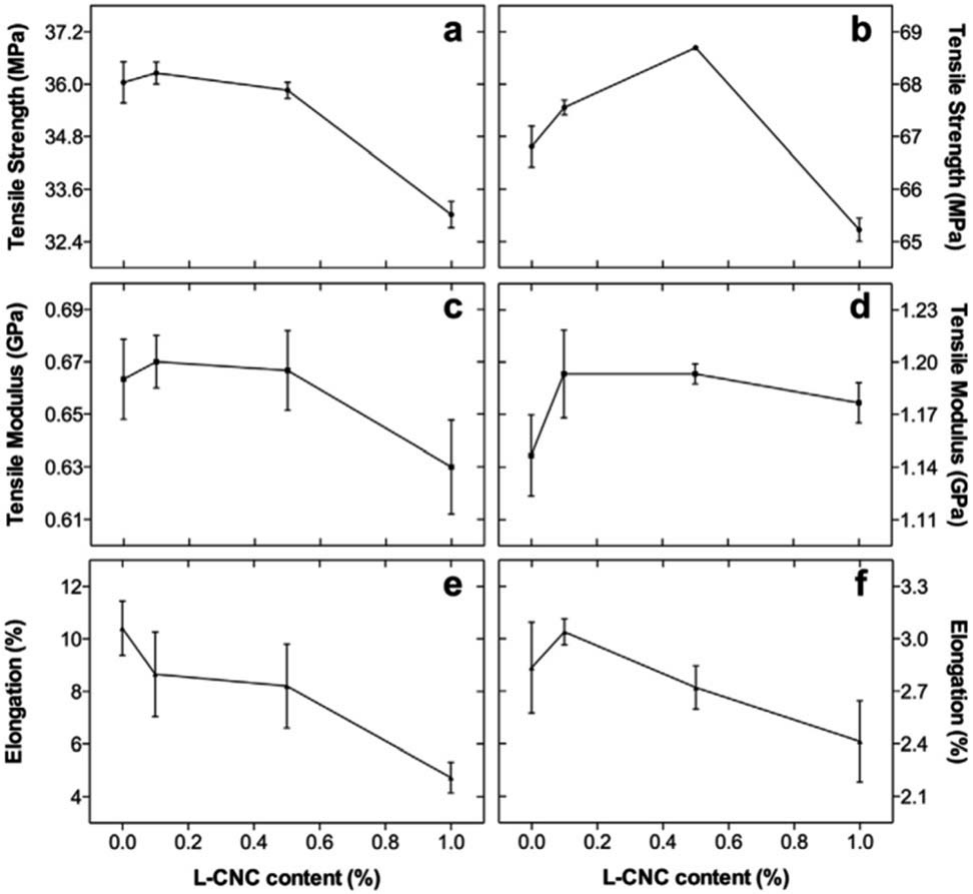
Mechanical properties (a and b) tensile strength, (c and d) tensile modulus, and (e and f) elongation of non-post-cured (a, c, and e) and post-cured (b, d, and f) 3D-ST printed L-CNC/MA nanocomposites (reproduced with permission from ref. 82. Copyright 2017, Elsevier).

In another study, Feng *et al.* reported the post-curing of lignin-coated cellulose nanocrystal (L-CNC) filled methacrylate-based nanocomposites and investigated their mechanical properties and thermal stabilization. Some small gaps or voids were found between the nanofiller and polymer matrix; however, these gaps decreased after the thermal post-curing at 120 °C for 40 min, where 0, 0.1, 0.5, and 1 wt% concentration of nanofiller was used in this study. In terms of mechanical properties, both the tensile strength and Young's modulus increased with the addition of 0.1 wt% L-CNCs. However, beyond 0.1 wt%, the mechanical properties were reduced by 5–8%. This was due to the insufficient interfacial dispersion caused by the agglomeration of L-CNCs in the methacrylate matrix, which was in accordance with many studies, where increasing the nanofiller concentration beyond its percolation threshold results in a decrease in mechanical properties.^122^ After post-curing, the addition of 0.1 and 0.5 wt% L-CNCs enhanced the mechanical properties and also improved the thermal stability, as shown in Fig. 14.^82^

#### 6.2.3 2-Dimensional nanoparticles/sheets

2-Dimensional sheets have at least one dimension in the nm scale. In terms of 2-dimensional nanofillers, graphene-based materials are most widely used in VPP-based 3D printing such as GNP (graphene nanoplatelets), graphene oxide (GO), and reduced graphene oxide (rGO). Graphene is a single layer sheet of carbon with sp^2^ bonding. Graphene is considered to be the strongest material in the world and best conductor of heat and electricity. Alternatively, GO is an oxidized form of graphene, with abundant oxygen-containing functional groups. Due to these excess functional groups, GO is easily dispersed in water and other solvents such as acetone and ethanol. Thus, because of these properties, most of the literature has multiple studies on GO in VPP for different applications.

In the preparation of polymer nanocomposites, mostly a solvent is used for the dispersion of the nanofiller and polymer matrix. However, removal of the solvent from the resulting composite is necessary, where even small traces of solvent can significantly affect its properties. Therefore, to overcome this issue, Feng *et al.* proposed an effective solvent-free technique for the fabrication of graphene-reinforced nanocomposites using DLP. The quick curing capability of this method allowed for a very homogenous nanofiller dispersion without the evident agglomerates that may result from the previously reported drawn-out curing of resins. Consequently, ‘the mechanical properties of the graphene-reinforced nanocomposites were significantly enhanced with just 0.5 wt% graphene, resulting in a 14% increase in flexural modulus and 28% increase in fracture toughness. A jawbone with a square architecture and a gyroid scaffold for bone tissue engineering applications were successfully constructed. Using the developed graphene-reinforced resins and DLP, they demonstrated the enormous potential of the current UV-curable nanocomposite resin systems for various applications in fields such as bioengineering.^81^

**Fig. 15:**
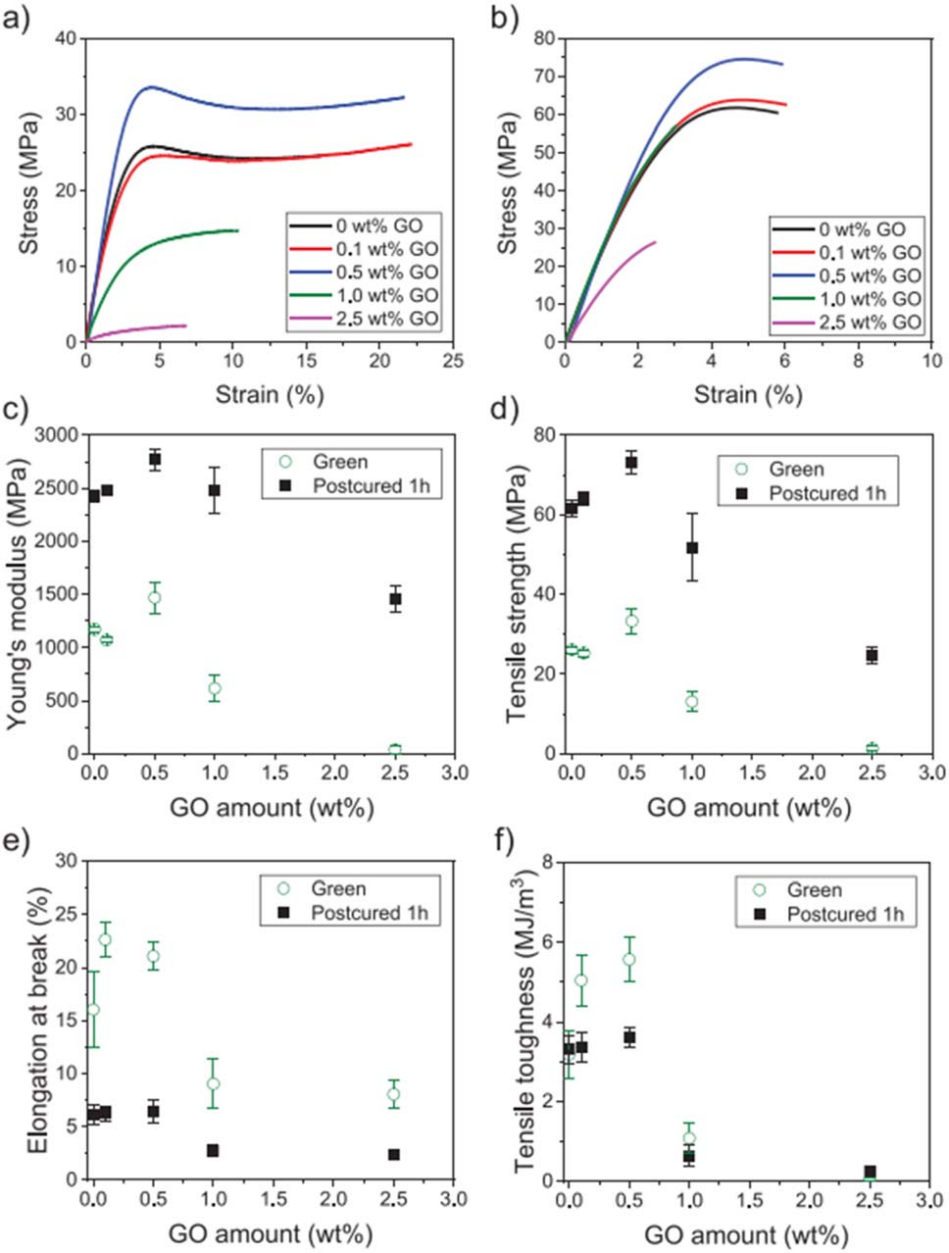
Representative tensile testing curves of nanocomposites containing 0–2.5 wt% GO: (a) after printing (green samples) and (b) after post-curing with UV for 60 min (post-cured samples). Average (c) Young's modulus, (d) tensile strength, (e) elongation at break, and (f) tensile toughness of the nanocomposites (reproduced from ref. 127. Copyright 2020, MDPI).

León *et al.* also studied the post-processing of 3D-printed polymer nanocomposites and performed a comparative study with samples having no post-processing involved. Different amounts of graphene (GNP) were mixed in acrylic-based photo polymer resin ranging from 0.5–5 wt%. Their study revealed that even though the nanofiller was well dispersed in the polymer matrix, the degree of cure and mechanical properties were worse compared to the control specimens. The tensile strength and Young's modulus decreased proportionally when the GNP content increased and the elongation at break decreased from 35% to 7–9% strain. However, when thermal post-processing was done on the samples at 60 °C with UV light for 1 h, a significant enhancement by about 90% was achieved in all properties. The mechanical properties were enhanced with up to 1.0 wt% GNP content, and then decreased due to the agglomeration of GNP in the resin. After thermal post-curing, the Young's modulus and tensile strength significantly increased from 1280 MPa and 48.8 MPa to 1880 MPa and 54.8 MPa, respectively, for the 0.5 wt% GNP nanocomposite, as shown in Fig. 15. The Young's modulus also increased to 1600 MPa for the 1.0 wt% GNP nanocomposite. Also, it was noted that post-curing beyond one hour had no effect on the mechanical properties of the nanocomposites. Similarly, electrical properties were also affected by post-curing. Interestingly, a huge increase of nine orders of magnitude was observed when the nanofiller content was increased up to 2.5 wt%, which was in the acceptable conductivity range of 10^−6^ S cm^−1^. The sheet resistance value was 10^7^ ohm sq^−1^, which was in the range of semiconductor dissipative materials (10^7^–10^9^ ohm sq^−1^). This value makes the nanocomposite a good material for preventing electrostatic discharge (ESD) problems.^127^

When dealing with printed polymer nanocomposites, there are a few issues. For instance, their mechanical properties increase when the amount of nanofillers in the polymer matrix increases up to a certain point, but they gradually decrease as the amount of nanofillers is increased further.

**Fig. 16:**
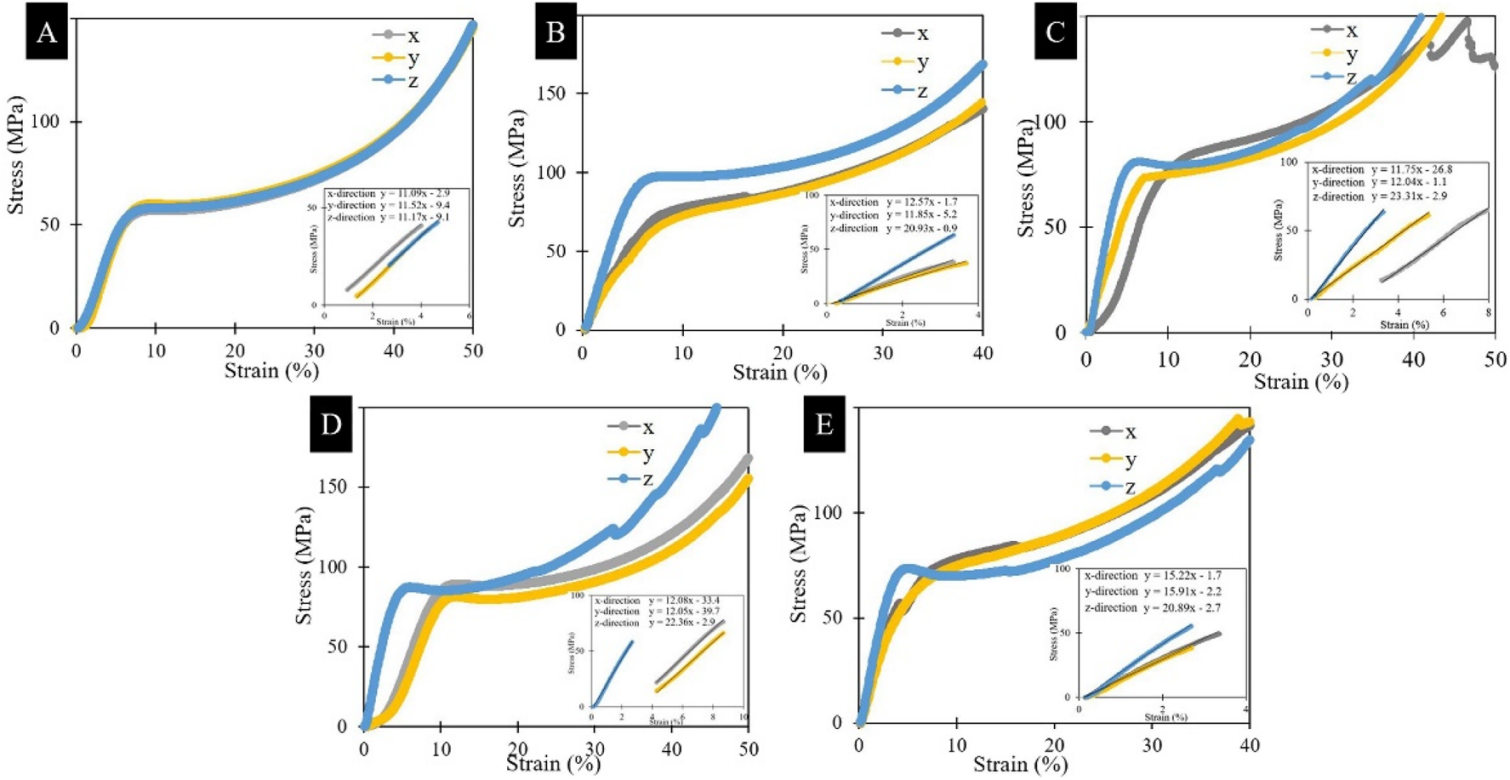
(A–C) Stress–strain curves of graphene polymer composites in different directions with increasing graphene concentration of (A) 0 wt%, (B) 0.01 wt%, (C) 0.02 wt%. (D) 0.05 wt%, and (E) 0.1 wt% (reproduced and adapted with permission from ref. 47. Copyright 2020, Elsevier) (for the interpretation of the references to colour in this figure legend, the reader is referred to the web version of this article).

Markandan *et al.* investigated the post-treatment issues and studied the mechanical properties of graphene-based nanocomposite lattice structures at very low graphene concentrations. Different parameters that affect mechanical properties were studied in detail such as nanofiller concentration, solvent addition and post-fabrication baking temperature. Different graphene concentrations (0.01–0.1 wt%) were used in this study. It was found that increasing the graphene concentration beyond 0.05 wt% resulted in significant agglomeration, which had a direct effect on the mechanical properties. Anisotropic behavior was observed across all the graphene–polymer composites. The Young's modulus and yield stress of the composite reinforced with 0.1 wt% graphene decreased by 72.4% and 17%, respectively, when the filler was dispersed in acetone, as shown in Fig. 16. Similarly, the mechanical properties increased with thermal post-curing, as evidenced by ref. 47, 72, 82, 122, 127, 129 and 132 It was observed that the Young's modulus and yield strength in the linear regime were more rapid in graphene nanocomposites compared to the control specimens. The Young's modulus and yield strength increased from 1601 MPa and 77 MPa to 2180 MPa and 88 MPa, respectively, compared to the control specimens. These exceptional mechanical properties of the SLA-based nanocomposites with very low graphene concentrations indicate their potential use in various applications such as aerospace, automotive and sports equipment.^47^

Korhonen *et al.* investigated graphene-based 3D structures by SLA 3D printing. Initially, they used a GO nanofiller to print the nanocomposite resin but later the printed sample was pyrolyzed to reduce graphene oxide (rGO) to entirely graphene to make it electrically conductive. This resulted in high shrinkage and brittleness in the 3D-printed parts. Electrical conductivities in the range of semiconductors were attained by partially pyrolyzing the polymer components, and the samples displayed superior dimensional stability and greater mechanical strength than the structures made entirely of graphene.^125^

The weak thermo-mechanical properties of 3D-printed polymers limit their applications in VPP-based processes. Thermal post-treatment can efficiently enhance the mechanical properties of the resulting polymer nanocomposites, as shown in many studies.^47,72,82,122,127,129^ It has been proven experimentally by many researchers that post-curing in the form of thermal or UV light enhances the mechanical properties of 3D-printed nanocomposites.

Lin *et al.* investigated the effect of post-curing and treatment on graphene-based nanocomposites for the first time using SLA 3D printing. They added 0.2 and 0.5 wt% GO to a commercially available resin and studied the effects of heat treatment post-curing. The tensile strength showed an enhancement of 24.9% and 45.3% for 0.2 wt% and 0.5 wt% of GO, respectively, compared to the control specimens. However, when the specimens were thermally treated in a furnace, their mechanical properties improved drastically with a tensile strength enhancement of 62.2%. Their results revealed that the increase in strength and ductility was related to the increase in the crystallinity of the nanofiller-reinforced composite.^79^

**Fig. 17:**
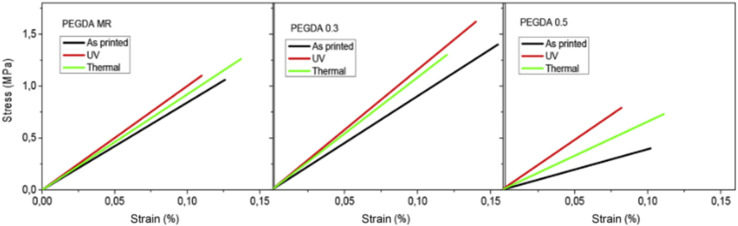
Stress–strain curves obtained for the PEGDA MR, PEGDA 0.3 and PEGDA 0.5 samples after different treatments (reproduced with permission from ref. 129. Copyright 2017, Elsevier).

**Fig. 18:**
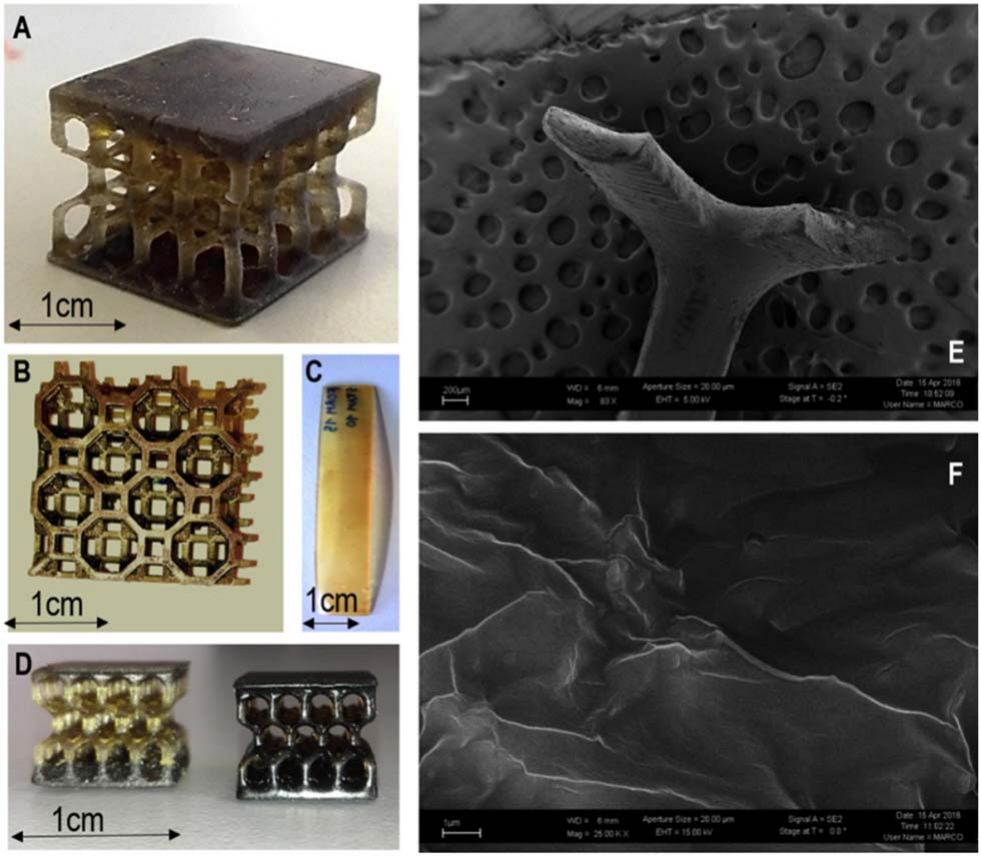
(A) Honeycomb structure used for compression tests produced with formulation PEGDA 0.5. (B) Complex geometry produced with the formulation PEGDA 0.3. (C) Specimen for tensile tests produced with the formulation PEGDA MR. (D) Color change of small honeycomb structures in PEGDA 0.5 before (left) and after (right) UV post-treatment. (E and F) SEM images of a section of a honeycomb structure in PEGDA 0.5 at different magnifications (reproduced with permission from ref. 129. Copyright 2017, Elsevier).

It is evidenced from many studies that post-curing promotes the cross-linking of the free monomers in the polymer matrix, which creates new covalent bonds that increase the strength and stiffness.^47,72,82,122,127,129,132^ Chiappone *et al.* showed that the thermal and UV post-curing of graphene oxide-based 3D printed nanocomposites can effectively enhance the thermal, electrical and mechanical properties of the resulting nanocomposites, as shown in Fig. 17 and 18, respectively.^129^

León *et al.* investigated the thermal post-curing of graphene oxide (GO)-based nanocomposites by SLA 3D printing and investigated their effect on mechanical properties. Increasing the nanofiller concentration up to 1 wt% resulted in an increase in mechanical properties; however, beyond 1 wt%, these properties decreased rapidly. After thermal post-curing, the tensile strength and modulus increased by almost 100% for 0.5 wt% GO compared to the control specimens.^122^

**Fig. 19:**
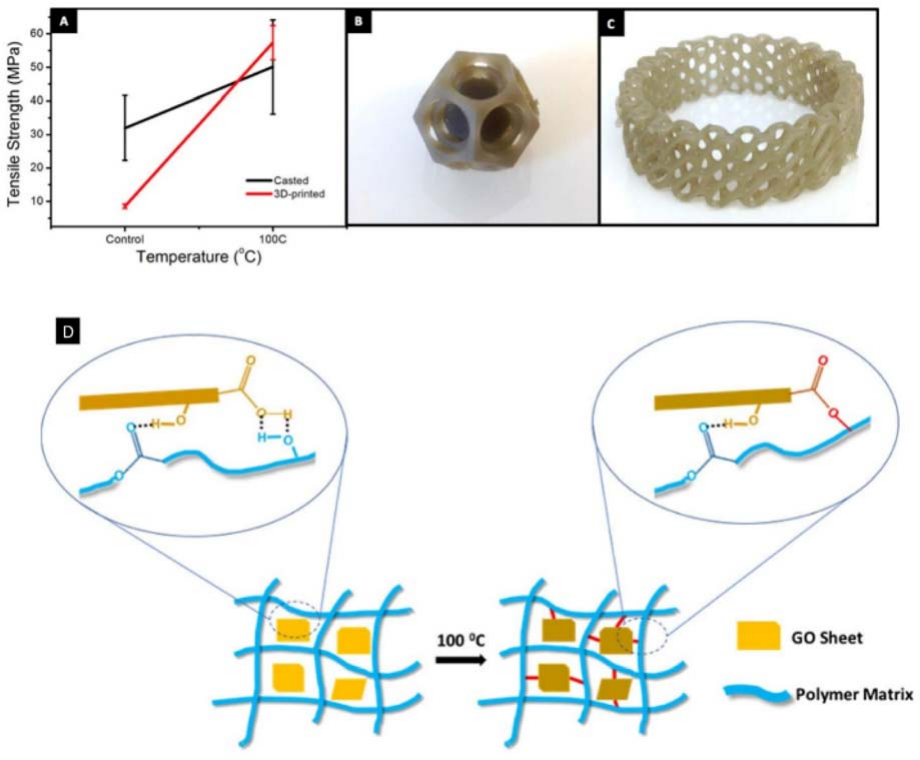
(A) Comparison of the tensile strength of the casted and 3D printed parts. SLA-printed complex structures of (B) nested dodecahedron and (C) diagrid ring. (D) Covalent and non-covalent interaction of 3D-printed GO nanocomposites (reproduced and adapted with permission from ref. 126. Copyright 2017, the American Chemical Society).

Manapat *et al.* also addressed this issue and post-cured the nanocomposites, which resulted in an enhancement in mechanical and thermal properties. A small amount of GO nanofillers was added to a commercially available SLA resin for reinforcement, and it was thermally annealed for 12 hours at 50 °C and 100 °C. At an annealing temperature of 100 °C, tensile testing showed an increase in the strength and modulus, with the maximum tensile strength increase recorded at 673.6% (for 1 wt% GO), as shown in Fig. 19. This enhancement was due to the enhanced cross-linking and decrease in the pore size of the resin with an increase in the annealing temperature. Both differential scanning calorimetry (DSC) and thermogravimetric analysis (TGA) demonstrated the increased thermal stability with an increase in the annealing temperature. The metastable structure of GO, polymer–nanofiller cross-linking through acid-catalyzed esterification, and elimination of intercalated water were all credited for the tremendous improvement in mechanical properties.^126^

The dispersion of nanofillers in the polymer matrix plays an important role in the mechanical and thermal properties of 3D printed nanocomposites. Thus, Tsang *et al.* studied this behavior in GO-modified flexible polymer nanocomposites and noted that an increase in nanofiller concentration affects the mechanical and thermal properties. The mechanical strength, stiffness, elongation and thermal properties of the resulting nanocomposites decreased with the addition of GO. This phenomenon is in accordance with other studies that GO agglomerates inside the resin affects its properties.^47,48^ Future development of 3D-printed nanocomposite elastomers was suggested after more thorough research on efficient methods to create homogeneous GO dispersions in SLA resins and the heat treatment of the nanofiller/elastomer nanocomposites for mechanical and thermal applications.^78^

Agglomeration is a serious problem in dealing with nanocomposites. It is more evident that if the filler concentration increases, it will not properly be dispersed inside the matrix, which results in a decrease in Young's modulus. This is due to the weak interface between the filler and matrix. To help reduce the agglomeration of nanoparticles, their functionalization and proper optimization of their preparation routes can alleviate this problem.^48,59^

**Fig. 20:**
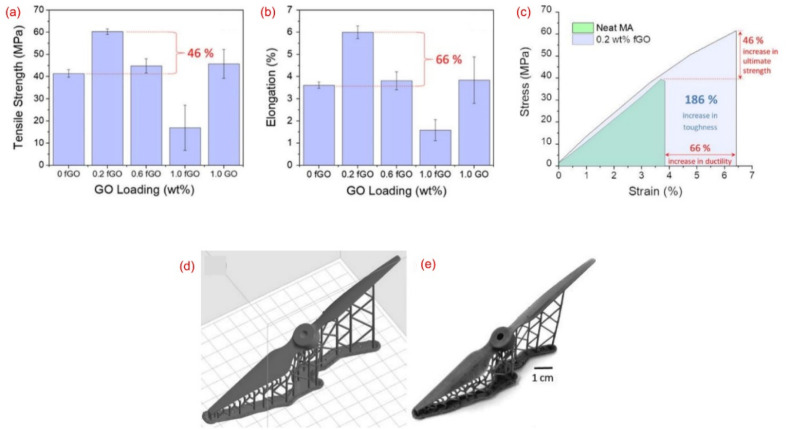
(a) Tensile strength. (b) Elongation of 3D-printed specimens as a function of filler concentration. (c) Stress–strain curves of neat polymer and 0.2 wt% fGO. (d) .stl format of the part to be printed and (e) real 3D-printed part suitable for drone parts manufacturing. In comparison to neat polymer, 0.2 wt% fGO resulted in 66% increase in ductility and 46% increase in tensile strength with the overall toughness increase by 186% (reproduced and adapted with permission from ref. 48. Copyright 2019, ACS).

Functionalized graphene oxide (fGO) nanocomposites are suitable materials for numerous applications, including the production of drone parts, due to their exceptional tensile properties. Palaganas *et al.* printed an fGO nanocomposite by SLA to enhance its mechanical and thermal properties with high quality, printing repeatability, and mechanical integrity. Different concentrations of functionalized nanofiller (0–1.0 wt%) was mixed with commercially available methacrylate polymer resin. The addition of fGO up to 0.2 wt% enhanced the tensile strength and ductility by 46% and 66%, respectively. However, increasing the nanofiller concentration beyond 0.2 wt% degraded the mechanical properties. This phenomenon is in accordance with the principle of the percolation threshold. The production of homogenously distributed fGO in various polymer matrices, which produces high interfacial adhesion due to the efficient transmission of a load from the matrix to the filler, is another significant advancement in the study. The ability of GO to form covalent bonds with different polymer matrices through functionalization to maximize its mechanical properties, together with its well-known good antimicrobial properties, make it a potential material for medical applications such as its use in external and internal medical devices and drone parts manufacturing, as shown in Fig. 20.^48^

#### 6.2.4 Factors affecting mechanical properties

Uncontrolled anisotropy is a huge problem in AM technologies in achieving the desired mechanical properties.^32^ Although the anisotropy in SLA and DLP is less than that in FDM and other methods, it still needs to be considered. If the part is produced with no or little understanding of anisotropy, inconsistency in terms of mechanical properties can be observed. Similarly, post-processing affects the mechanical properties.^47,127,132,133^ The mechanical properties of the printed parts can be significantly improved by UV post-curing, because of the complete curing of the uncured resin inside the part.^133^ UV-curing and thermal curing are used in post-processing. In terms of effectiveness, UV-post-curing is more effective than thermal curing in increasing mechanical properties.^32,132^ The post-processing method and duration should be carefully selected and optimized to achieve the maximum mechanical properties.^112^ The layer thickness, print resolution, build orientation, *etc.* also affect the mechanical properties. In fact, the layer thickness can be related to the tensile and impact strength according to the following relation^32^Layer thickness ∝ tensile strength/impact and flexural strength

The intensity of the light source also affects the mechanical properties of a printed part. A light source with a wavelength of 405 nm produces parts with excellent mechanical properties in comparison to a light source with a low wavelength.^133^ If there are no equipment and material constraints, then a 405 nm light source is best to achieve the maximum properties. Given that there are lots of variations in mechanical properties by different AM methods, it is not possible to make a single standard for mechanical tests that is followed by all methods but there is still a need for a set of rules to standardize the mechanical testing or characterizations of 3D-printed polymer parts made by VPP.^1,32^

### 6.3 Thermal, magnetic and electrical applications

The VPP process has great potential in printing magnetic nanocomposites compared to FDM.^134^ Their applications include sensors, micro-actuators, and other health-related applications.^135–137^ Different magnetic nanofillers are used in photopolymer resins to increase their mechanical and magnetic properties, *etc.*^138^ Iron oxide is the most widely used nanofiller in the literature.^50,52^ Some of the magnetic fillers studied and their resulting applications in the past three years are summarized in Table 4.

**Table 4:** Different polymer nanocomposites produced by VPP processes for thermal, magnetic and electrical applications

Base material + nanofiller	Additives and reinforcements	Highlights	Properties enhanced	Applications	Ref.
UV curable resin + MWCNTs	MWCNT conc. (up to 0.6 wt%) urethane acrylate 57.7%, acrylate monomer 38%, photoinitiator 4%	Layer thickness at *z*-axis (25–100 μm)	Electrical conductivity achieved 0.071 S m^−1^	Capacitive sensors, mechanical interfaces	139
		Light wavelength 405 nm	High dimensional accuracy at 0.6 wt%		
		Resolution in *XY* plane 47 μm			
Self-prepared resin + graphene + Fe_3_O_4_	G conc. (0.30 g)	Light intensity: 13 mW cm^−2^ light source (385 nm)	Maximum achieved Young's modulus at 0.8 wt% was 3.1 GPa at 0° angle	Electrical components	140
	Fe_3_O_4_ (0.10 g)	UV curing 4 s	Maximum achieved Young's modulus at 1.6 wt% was 2.6 GPa		
	Tricyclodecane dimethanol diacrylate (DCPDA) (monomer)	Glass slide spacer thickness 100 μm	Percentage change in modulus was 4%		
	Phenyl bis(2,4,6-trimethylbenzoyl) phosphine oxide (TPO) (photoinitiator) sodium dodecylbenzene sulfonate (SDBS)				
Esun bio-based polyurethane resin + G	G conc. (0.5, 1, 2 wt%)	Resin density 1.07–1.13 g cm^−3^	0.5 wt% G reduced the friction co-efficient by 50%	Friction and wear	141
	Acrylate 30% min	Viscosity of 200–300 mPa s	Vertical and 45° angle orientations resulted in more than 56.5% growth in average surface roughness of 100 μm		
	Monomer 30% min	Light wavelength 405 nm	Surface roughness increased by 74.4%		
	Photo initiators 5% max		Shore hardness increased as G conc. increased		
	Color pigment 5% max				
Methacrylate malate photocurable resin + CNCs	CNCs conc. (0.25, 0.5, 0.75, 1 wt%)	Exposure time 25 s	Maximum achieved capacitance improved by 132% with 1 wt% CNCs	Energy storage materials	142
	(Trimethylbenzoyl)phosphine oxide (TPO) 0.5 wt%	Layer thickness 0.05 mm	Maximum achieved dielectric constant was 10.9 at 1 MHz		
Acrylic resin + BaTiO_3_	BaTiO_3_ conc. (10, 20, 30, …, 90 wt%)	Exposure time 2.8 s	Maximum achieved bending strength was 57.9 MPa	Orthopaedics, bone implant materials	143
	Triethanolamine	Layer thickness 35 μm	Maximum compressive strength achieved was 70 MPa		
	Tetraethyl orthosilicate	Power density 90 mW cm^−2^	Maximum relative density was 94.2%		
	Camphor quinone	Optimal sintering temperature 1425 °C for 2 h	Highest piezoelectric coefficient achieved was 241 pC N^−1^		
	Benzoate				
HDDA-based commercial UV-curable resin (named JA-01) + GNP	GNP conc. (1, 2, 3, 4 wt%)	Light intensity of 37.49 mW cm^−2^	Increase in thermal conductivity by up to 14.6%	Heat sinks	144
	Phenylbis(2,4,6-trimethylbenzoyl) phosphineoxide (BAPO)	Exposure time 5 s	Maximum tensile strength achieved was 14.9 MPa		
	4,4′-Diaminodiphenylmethane (DDM)		Elongation at break was 8%		
Acrylic photopolymer resin + BaTiO_3_	BaTiO_3_ conc. (70, 75, 80, 82, 84, 86 wt%)	Sintering temperature 1290 °C	Maximum piezoelectric coefficient achieved was 166 pC N^−1^ at 80%	Ultrasound transducers	143
			Electrochemical coefficient range was 0.73–0.416		
Bisphenol A ethoxylate diacrylate (SR349) + Fe_2_O_3_	Fe_2_O_3_ conc. (1, 1.5, 2, 4 wt%)	Penetration depth < 50 μm	Theoretical Young's modulus was 23 GPA	Magnetic sensors, MEMS relays	51
	2,4,6-Trimethylbenzoylphenyl phosphinate (PI)	Resin viscosity 1500−2000 mPa s at 25 °C	Spring constant was 0.16 N m^−1^		
	Oracet yellow 130 visible dye	Light wavelength 405 nm			
		Power output 30 MW			
		Laser line spacing 0.02 mm			
EAA (EBECRYL 8413) and aliphatic urethane diacrylate with 33 wt% of isobornyl acrylate resin + MWCNTs	MWCNT conc. 2 to 5 wt% isobornyl acrylate	Light wavelength 405 nm	High mechanical durability (10 000 cycles), highest sensitivity of 8.939, linearity up to strain of 45%	Human motions	145
	(Trimethylbenzoyl) phosphine oxide (TPO)	Power output 30 MW	Strain detection range of ≈60% and low detectable limitation of 0.01%		
Ebecryl 8232 a urethane-acrylate resin + Fe_3_O_4_	Fe304 conc. (0–8 wt%)	Light intensity 10 mW cm^−2^	Maximum tensile strength achieved was 7 MPa	Soft robotics, flexible electronics	52
	Phenylbis(2,4,6-trimethylbenzoyl)phosphine oxide (PI)	*XY* resolution 50 μm	Tensile modulus decreased up to 6 wt%		
	Butyl acrylate conc. (0, 25, 50 wt%)	*Z* resolution 10 μm			
		UV post-curing 10 min			
600 clear (E-Shell), ABS 3SP tough (ABS) + Ferucarbotran (FCT)	Acrylic resin conc. (60–80%)	*XY* resolution 42 μm	MPI performance was 5.6 mg mL^−1^	Magnetic particle imaging	53
	Urethane dimethacrylate (5–20%)	Projector resolution 1920 × 1200 pixels			
	Tetrahydrofurfuryl methacrylate (10–25%)	*Z* resolution range 25–150 μm			
	Diphenyl (2,4,6-trimethylbenzoyl) phosphine oxide (less than 1%)				
Poly(ethylene glycol) diacrylate (PEGDA) + poly(3,4-ethylenedioxythiophene) polystyrene sulfonate (PEDOT:PSS)	Irgacure 819 (PI) 0.5, 1, 6 wt%	Printer (Microla Optoelectronics)	Maximum electrical conductivity achieved was 0.05 S cm^−1^	Biosensors, energy storage	146
	PEDOT:PSS 1, 5 wt%	Laser power density 15 W mm^−2^	Maximum Young's modulus achieved was 21 MPa		
		Scan velocity 1000 mm s^−1^			
		Hatch spacing 50 μm			
		Layer thickness 100 μm			
Monofunctional EAA (EBECRYL 113) and AUD (EBECRYL 8413) + rGO	rGO conc. 1, 2, 3 wt%	Printer B9 Core 550, B9 Creations	Highest sensitivity of 6.723 at linear detection range of 40%	Strain sensors, human motion detection	147
	AUD and EAA ratio 1 : 9	Light wavelength 405 nm	High mechanical durability (10 000 cycles)		
	Diphenyl (2,4,6-trimethylbenzoyl) phosphine oxide (TPO)	Power output 200 W			
		Light intensity 30 mW cm^−2^			
Conduct-O-Fil AG CLAD 12 acrylate + silver	Silver conc. 1.0–4.0 wt%	Work again on this reference	Electrical conductivity range was 31–793 S m^−1^	EMI shielding, flexible electronics	138
Poly(ethylene glycol) diacrylate + CNTs	CNTs conc. (0.1, 0.3, 0.5, 1, 1.5 wt%)	Light wavelength 405 nm	Maximum Young's modulus achieved was 10.3 MPa	Electrical components	148
	Bis(2,4,6-trimethylbenzoyl)-phosphineoxide (BAPO) (PI)	Light intensity 22 mW cm^2^	Maximum conductivity achieved was 8.00 × 10^−6^ S cm^−1^		
		Exposure time 2–3 s per layer			
Genesis® Development resin	G conc. up to 2 wt% carbonyl iron	Light wavelength 406 nm	Maximum reflection loss was −54.4 dB	Microwave absorbers	149
Base (Tethon 3D) + graphene		Light intensity: 33.4 mW cm^−2^	Effective absorption bandwidth was 3.41 GHz	Rapid prototyping	
		Slice thickness: 50 μm			
		Exposure time: 3, 5, 8, and 12 s			
3DSR ultra-high resolution resin + iron oxide (Fe_3_O_4_)	Fe_3_O_4_ conc. 0.5 wt%	Titan2 SLA printer	Relative grayscale value achieved was 70	Magnetic sensors, biomedical implants	150
	Isobornyl acrylate (diluent)	Layer thickness 100 μm	Highest magnetic flux density was Norm 1.00 × 10^−4^ T		
		Exposure time 8–5 s			
High temp resin V1 (Formlabs) + CNTs	CNTs conc. (0.030, 0.050, 0.075, 0.100, 0.150 wt%)	B9Creator printer	Maximum electrical conductivity achieved was 1 × 10^−2^ S m^−1^	Strain sensing applications	151
		Layer thickness 30 μm	Maximum Young's modulus achieved was 2.9 GPa		
		Exposure time 5.12–6.84 s			
Flex resin (Makerjuice Lab) + PZT	PZT conc. 2, 5, 10, 18 wt%	Layer thickness 25 μm	Maximum dielectric permittivity was 120 at 100 Hz	Electronic applications, capacitors	152
	Methacrylate 60–90 wt%	Exposure time 10 s	Dielectric loss was 0.028 at 100Hz		
	1,6-Hexanediol methacrylate 5–15 wt%		Maximum capacitance was 63 Fg^−1^ at a current density of 0.5 Ag^−1^		
	Photoinitiator 0.1–1 wt%				
Grey resin (Formlabs) + graphene	G conc. 0.02, 0.05, 0.10 wt%	Form 2 Formlabs printer	Maximum tensile strength achieved was 175 MPa	Lattice structures	58
		Light wavelength 405 nm	Maximum modulus was 65 MPa		
		Layer resolution 0.05 mm	Maximum energy absorption was 14 kJ m^−3^		
		Printed material density 1.20 g cm^−3^			
TEGORAD 2800 (TRAD), a PDMS acrylate + boron nitride (BN)	BN conc. (0, 0.99, 2.91, 4.76 wt%)	Asiga printer	Maximum conductivity achieved was 0.21 W mK^−1^	Electronics	153
	Bis(2,4,6-trimethylbenzoyl)-phosphineoxide (BAPO) (PI)	Light wavelength 405 nm	Maximum tensile strength achieved was 1.24 MPa		
		*X*–*Y* resolution 50 μm	Maximum elongation at break achieved was 40%		
		*Z* axis control 1 μm			
		Layer thickness 50–75 μm			
		Light intensity 40 mW cm^−2^			
		Exposure time 10–25 s			

**Fig. 21:**
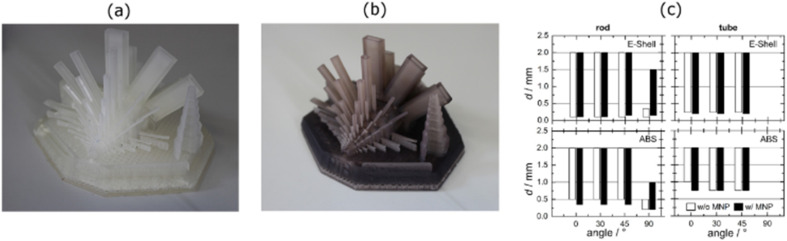
3D-printed demonstrator using E-shell without MNP (a) and with MNP (b) at a concentration of *c*(Fe) = 3.5 mg mL^−1^. The evaluation of the process ability of two different photopolymers (E-shell and ABS) with and without MNP (EFH3) was done by visual control of each structure. For each angle, with respect to the printing direction (0°, 30°, 45°, and 90°), the size range in which a successful formation was observed is covered by the bar (c) (reproduced from ref. 53. Copyright 2019, Elsevier).

Löwa *et al.* investigated the feasibility of magnetic nanocomposites for the manufacturing of sophisticated magnetic particle imaging (MPI) sensors and phantoms, as shown in Fig. 21. A protocol was developed for the systematic quality evaluation of 3D-printed magnetic nanocomposites to fabricate long-term, complex MPI sensors with defined magnetic properties. To magnetically characterize the liquid resin and the printed photopolymers with embedded nanofillers, the quasi-static and dynamic magnetization behavior were measured.^53^

In another study, Lai *et al.* studied the viscoelastic and high strain rate response of anisotropic graphene-based polymer nanocomposites by dynamic mechanical analysis and split-Hopkinson pressure bar. Graphene nanoparticles were mixed in a commercial photopolymer resin with a concentration of 0.02 wt% to 0.10 wt%. The storage modulus and dynamic failure strength of the printed nanocomposites improved with an increase in nanofiller concentration up to 0.8 wt% due to the maximum load transfer to the nanofillers. However, increasing the concentration up to 0.10 wt% resulted in worse mechanical properties due to the presence of a weak interface between graphene and the polymer resin. This was due to the agglomeration of the nanoparticles in the polymer matrix. The resulting nanocomposites were also tested for different axes for the alignment of graphene in the polymer. When the nanocomposite was loaded in an axis parallel to the print axis, the composite adopted an isostrain geometry, which offered high dynamic modulus and strength. The printed nanocomposite lattice structures showed better energy absorption capabilities than balsa wood, syntactic ceramic foams and Al foams on a per unit weight basis.^58^

The strain-sensing performance of printed nanoparticles has been widely studied using the resulting nanocomposites. For example, Cortés *et al.* studied the mechanical and strain sensing capabilities of carbon nanotube (CNT)-based nanocomposites by DLP 3D printing. Different factors were analyzed including nanofiller concentration and post-curing. It was found that post-treatment significantly improved the Young's modulus and glass transition temperature, *T*_g_. The strain sensitivity was measured by tensile and bending tests. The strain sensing tests showed a linear response of electrical resistance with applied strain with greater sensitivity achieved by decreasing the CNT concentration. Also, the electrical sensitivity of the tensile tests was more than the bending tests due to the compression-subjected face effect. An increase in nanofiller concentration decreased the Young's modulus of the resulting nanocomposites due to the high shielding effect.^151^

**Fig. 22:**
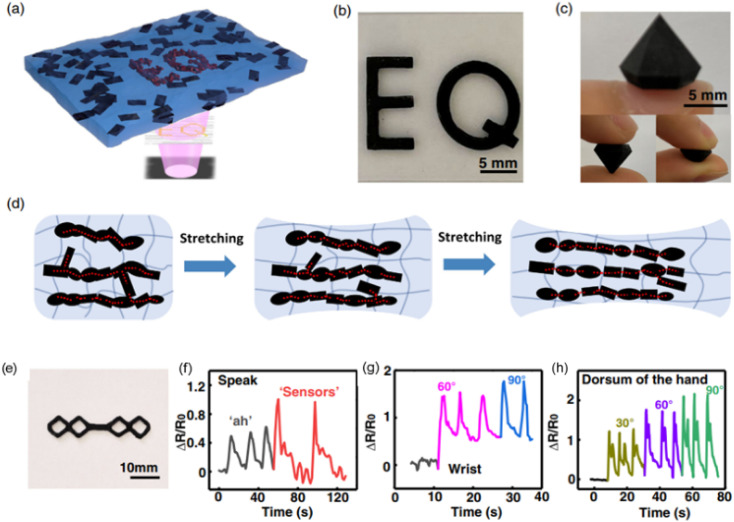
(a) Schematic diagram of the DLP-based 3D printing process. (b) Photograph of 3D-printed letters “E” and “Q” based on RGO/ER. (c) Photograph of RGO/ER-based diamond by DLP-based 3D printing. (d) Conductive path variation in the RGO/ER composite during mechanical deformation. (e) Photograph of an all 3D-printed flexible strain sensor based on RGO/ER. (f) Electrical responses of the strain sensor for pronunciation test. (g) Electrical responses of the device for the bending of the wrist. (h) Electrical responses of sensor under the bending of the dorsum of the hand (reproduced and adopted with permission from ref. 147. Copyright 202, Wiley Online Library).

Qian *et al.* printed a reduced graphene oxide rGO/elastomer nanocomposite resin with structural-modulated sensitivity for monitoring various human motions and flexible strain sensors by DLP 3D printing. The rGO/ER nanocomposite resin was prepared by mixing acrylated aliphatic epoxy (EAA), aliphatic urethane diacrylate (AUD), and rGO in a certain mass proportion. The nanofiller concentration had a direct effect on the electrical conductivity of the nanocomposite. The incorporation of less than 2 wt% rGO did not make the composite conductive, whereas above 2 wt% increased the viscosity, thus making it difficult for printing. In this case, 2 wt% rGO was used throughout the study. The resulting nanocomposite had a sensitivity of 6.723 at a strain rate of 40% and good stability and mechanical durability of more than 10 000 stretching–relaxing cycles, as shown in Fig. 22. A flexible strain sensor and rhombic structure as electrodes were printed, demonstrating its potential use for human motion detection.^147^

**Fig. 23:**
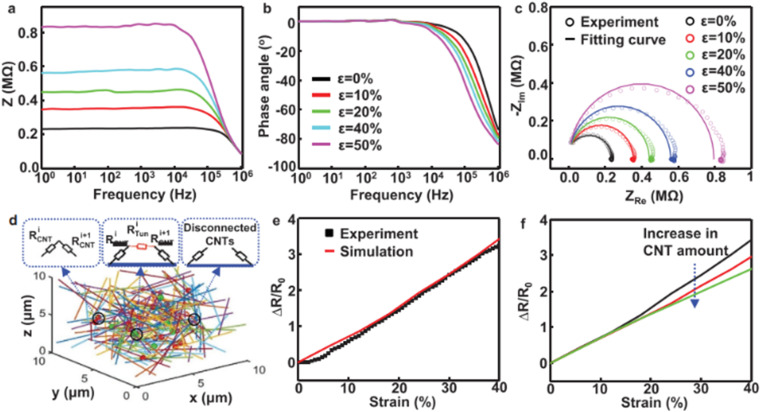
(a) Total impedance (*Z*)–frequency curves of the MWCNT/EA-2-based sensor at various strain. (b) Phase angle (*θ*)–frequency curves of the MWCNT/EA-2-based sensor at various strain. (c) Cole–Cole plots of the MWCNT/EA-2-based sensor at various strain and the fitting curve with an equivalent circuit model. (d) Schematic for the random distribution MWCNTs in a unit cell and the different interconnections between two MWCNTs. (e) Electrical response of the MWCNT/EA-2-based sensor to the applied strain by experimental measurement and numerical simulation. (f) Numerical simulation of relative resistance variation in response to the strain at different numbers of MWCNTs in a 3D unit cell (reproduced with permission from ref. 145. Copyright 2020, Online Wiley Library).

Xiao *et al.* developed flexible strain sensors by DLP 3D printing using UV-curable multi-walled carbon nanotube (MWCNT)-based nanocomposites. Different concentrations of MWCNTs (2–5 wt%) were used in this study. Increasing the concentration of MWCNTs increased the elastic modulus, which was attributed to the high stiffness of the nanofillers and their strong interfacial bonding with the polymer matrix. Similarly, to achieve the maximum dispersion of nanofillers, introducing a magnetic field in the printing process or using surfactant-encapsulated MWCNTs was suggested for future study. Accordingly, 2 wt% of the nanocomposite delivered a sensitivity of 8.939 with linearity of up to 45% strain. The strain sensor had a detectable strain range from 0.01% to 60%, high mechanical durability (10 000 cycles), and linear responses to humidity and temperature, as shown in Fig. 23. The 3D-printed flexible strain sensor arrays showed efficiency in detecting various external deformations.^145^

Safaee *et al.* investigated field-assisted DLP to print functionally graded nanocomposites embedded by magnetic nanoparticles. Black iron oxide (Fe_3_O_4_) nanoparticles were mixed in a commercial high-resolution polymer resin. The nanofiller concentration was fixed at 0.5 wt% together with a control sample having no fillers. Some parts of the printed specimens did not experience a magnetic field due to the strong aggregation of the nanoparticles. The concentration of the nanoparticles was dependent on the surface area and strength of the magnets as well as the distance between the printing bed and the magnets. A finite element model was also developed to verify the experimental results and the effect of magnet location on the nanoparticle concentration.^150^

**Fig. 24:**
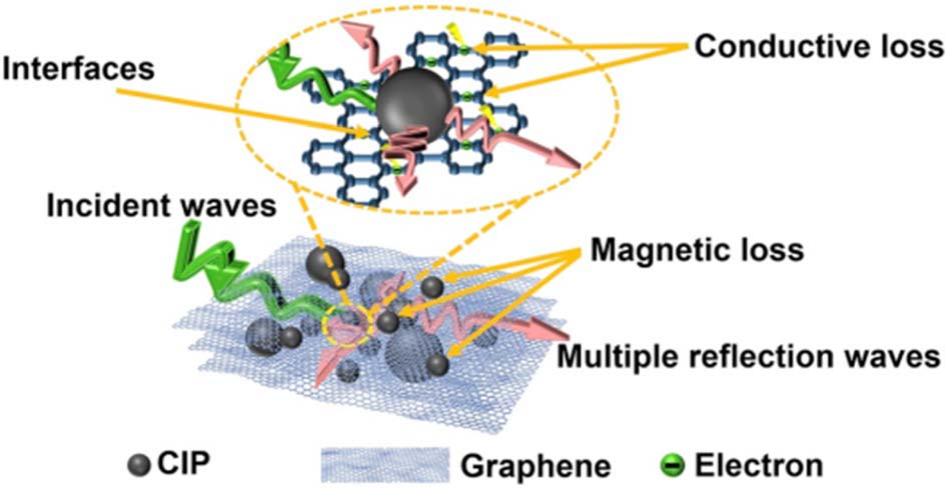
Schematic diagram of microwave absorption mechanism of graphene/CIP/PMMA composites (reproduced with permission from ref. 149. Copyright 2019, Elsevier).

Zuo *et al.* studied the microwave absorption characteristics of graphene-based methacrylate nanocomposites by DLP 3D printing. Different concentrations of graphene and carbonyl iron were used to adjust the resin composition to achieve the maximum properties. The nanocomposite produced with 1 wt% graphene had a maximum reflection loss (RL) of 54.4 dB at a thickness of 2.1 mm and an efficient absorption (RL: −10 dB) bandwidth as wide as 3.41 GHz. The magnetic loss of the microspheres and the many dielectric resonances from the interfacial polarization and accompanying relaxation were the underlying determinants of the enhanced microwave absorbing properties, as shown in Fig. 24. Thus, these nanocomposites can be used in multiple applications including microwave absorbers.^149^

**Fig. 25:**
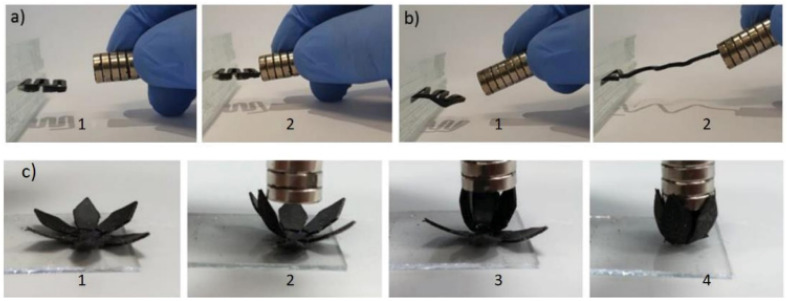
Different 3D printed magnetic objects. Spring printed with (a) 2 wt% filler concentration and (b) 6 wt% filler concentration. (c) Printed flower that closes when exposed to a magnetic field (reproduced and adapted with permission from ref. 52. Copyright 2019, Wiley Online Library).

Lantean *et al.* studied the magneto-responsive behavior of 3D-printed polymer nanocomposites with tunable mechanical and magnetic properties. The magnetic response of the printed structures could easily be altered with nanofiller loadings of up to 6 wt%. The stability of the nanofillers in the resin was also checked for printing as a result of their homogenous dispersion. Dynamic mechanical thermal analysis (DMTA) was used to examine the thermo-mechanical properties of the fabricated specimens. The mechanical properties of the magneto-responsive polymers were tailored from stiff to soft by combining acrylate resins with butyl acrylate and the magnetic response of the printed parts was tuned by changing the Fe_3_O_4_ loading. Different samples were printed and showed varying stiffness, and their magnetic response probed by rolling, translation, stretching, folding and unfolding, as shown in Fig. 25.^52^

Similarly, Credi *et al.* printed cantilever-based microstructures by using magnetite nanoparticles in the polymer matrix. High printing formulations were characterized by small critical energy and reduced penetration depth of less than 50 μm, which enabled fast printing of cantilever microstructures. Bisphenol A ethoxylate diacrylate (SR349) resin was used in this study. The magnetic sensitivity of the microstructures was characterized by tip deflection when an external magnetic field was applied magnetically actuated. The micro-sensing actuators were quantitatively studied by static flexural behavior *versus* the magnetic field applied. The results showed larger deformations for the nanocomposites printed entirely with ferromagnetic nanoparticles, and thus they can be potentially used in sensing applications.^51^

**Fig. 26:**
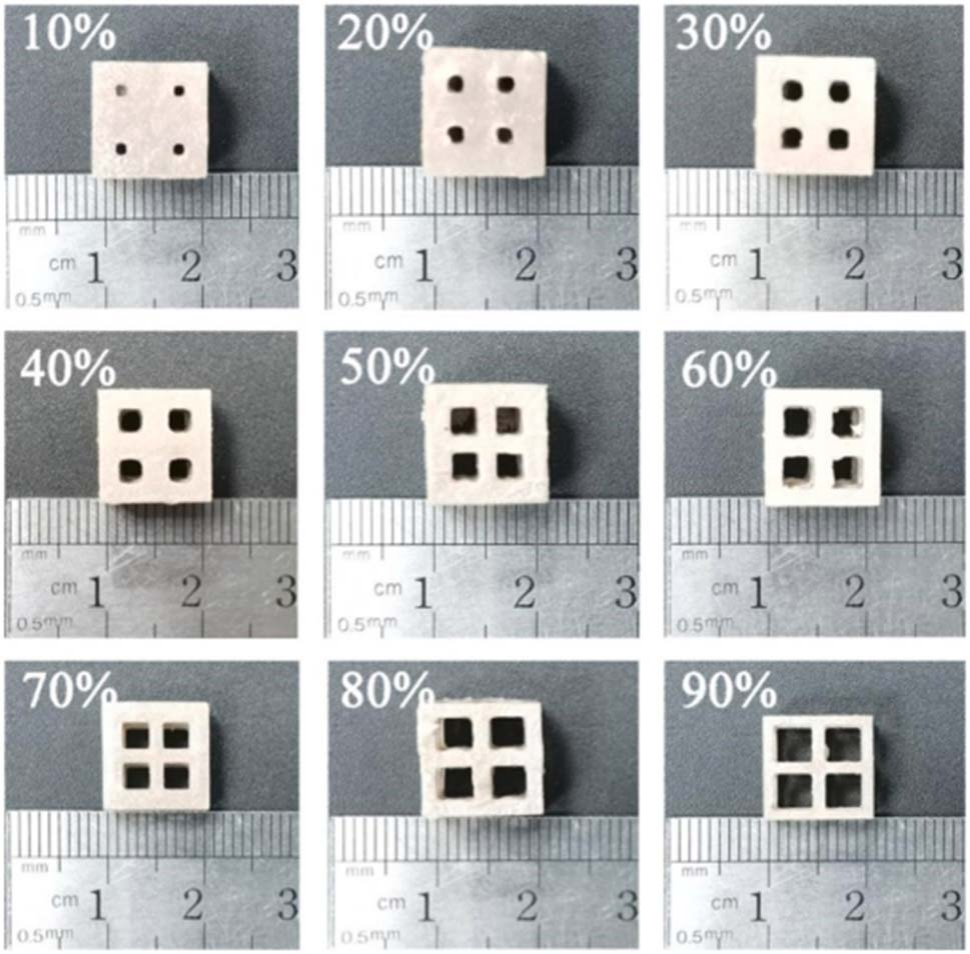
Porous structure of BaTiO_3_ ceramics and samples with different porosity (reproduced with permission from ref. 143. Copyright 2022, Elsevier).

In another study, Jiang *et al.* printed piezoelectric scaffolds using barium titanate (BaTiO_3_) nanoparticles and investigated their effects on mechanical and piezoelectric properties. The liquid photopolymer resin and different concentrations of BaTiO_3_ nanoparticles (10% and 20% up to 90%) were used to form different slurries. An increase in nanofiller concentration directly affected the density of the sintered nanocomposites. The electrical properties of the resulting nanocomposite were analyzed by printing ceramic disks with a diameter of 10 mm and thickness of 0.6 mm. The piezoelectric coefficient increased from 96 to 166 pC N^−1^ when 82 wt% content of nanofiller was used but then decreased beyond 82 wt%. By changing the porosity of the scaffold, the desired electrical and mechanical properties were achieved. An increase in porosity made the BaTiO_3_ scaffolds more uniform and easier to print. The maximum compressive strength piezoelectric coefficients were achieved at 10% porosity, as shown in Fig. 26. These nanocomposites have found applications in orthopedics and bone implants.^143^

Introducing a magnetic field inside the polymer resin affects different properties of the resulting nanocomposite. This idea was implemented by Younes *et al.*, who prepared a magnetic nanofiller-based nanocomposite, where the magnetic Fe_3_O_4_@graphene inside the polymer was aligned with a magnetic field. The mechanical properties of the nanocomposites were significantly enhanced by the alignment of the nanofillers and the angle of the magnetic field. It was evident from their results that 0° alignment of the nanofillers improved the mechanical properties more compared to 90° alignment at the concentrations of 0.4, 0.8, and 1.6 wt%. This enhancement was due to the excellent orientation and good dispersion of the nanofiller in the polymer matrix. The Young modulus showed a significant improvement at a concentration of 0.8 wt% and 0° magnetic field angle. An increase in the nanofiller concentration up to 1.6 wt% resulted in a decrease in the Young modulus despite the alignment of the filler materials. This was due to the incomplete polymerization caused by the aggregation of Fe_3_O_4_@graphene inside the polymer matrix, as evident somewhere, as too high concentration of nanoparticles blocks the incoming light source, which results in incomplete polymerization.^140^

The tribology of the nanocomposites was investigated by Hanon *et al.*, who studied the tribological characteristics of graphene-based nanocomposite by accessing different parameters such as the surface roughness, microstructure, friction, wear, and hardness properties of the tested materials. Here, in this study, low-concentration graphene contents in the polymer matrix were sufficient for the enhancement of the friction coefficient as it was reduced by 50% compared to the control samples. Furthermore, the impact of graphene on the tribological behavior of the nanocomposite and its difference with varying nanoparticle concentrations and different printing parameters were studied. Vertical and 45° angle orientations had substantial effects on the tribological properties.^141^

Recently, VPP processes such as SLA and DLP have found their way into many magnetic property-related applications, such as micro-actuators, fluidics, strain sensors, and functionally graded materials, *i.e.*, smart polymers, which are responsive to any external stimuli.^51,154^ Magnetic nanofiller-based composites printed with VPP are more feasible than that with the material extrusion and material jetting methods in terms of resolution, inhomogeneous magnetic response, and processing temperatures.^52^ Due to the ease of printability and the ability to build complex structures, VPP processes can be used for different smart applications using magnetic nanofillers. Some new modifications in the machine to stabilize the fillers inside the resin, investigating new materials as fillers and the matrix and proper optimization of the nanocomposite preparation routes are some suggestions for future research.

The electrical and thermal properties of polymer nanocomposites printed by VPP processes are also studied in parallel. There are many factors that affect their electrical and thermal conductivity, including filler concentration and matrix type.^155^

Pezzana *et al.* printed polydimethylsiloxane (PDSM)-based polymer nanocomposites with enhanced thermal conductivity using boron nitride nanofillers by DLP 3D printing. Before printing, the viscosity and reactivity of different formulations were checked. It was found that with an increase in nanofiller concentration, the viscosity increased and a delay in crosslinking occurred. The viscoelastic and thermomechanical properties of the 3D-printed nanocomposites had a slight effect on the *T*_g_, and an increase in the elastic modulus of the polymer matrix occurred with an increase in the nanofiller concentration. Similarly, the thermal conductivity showed an improvement in terms of *k* values with an increase in the filler by about 46% and the thermal diffusion had a 78% enhancement.^153^

**Fig. 27:**
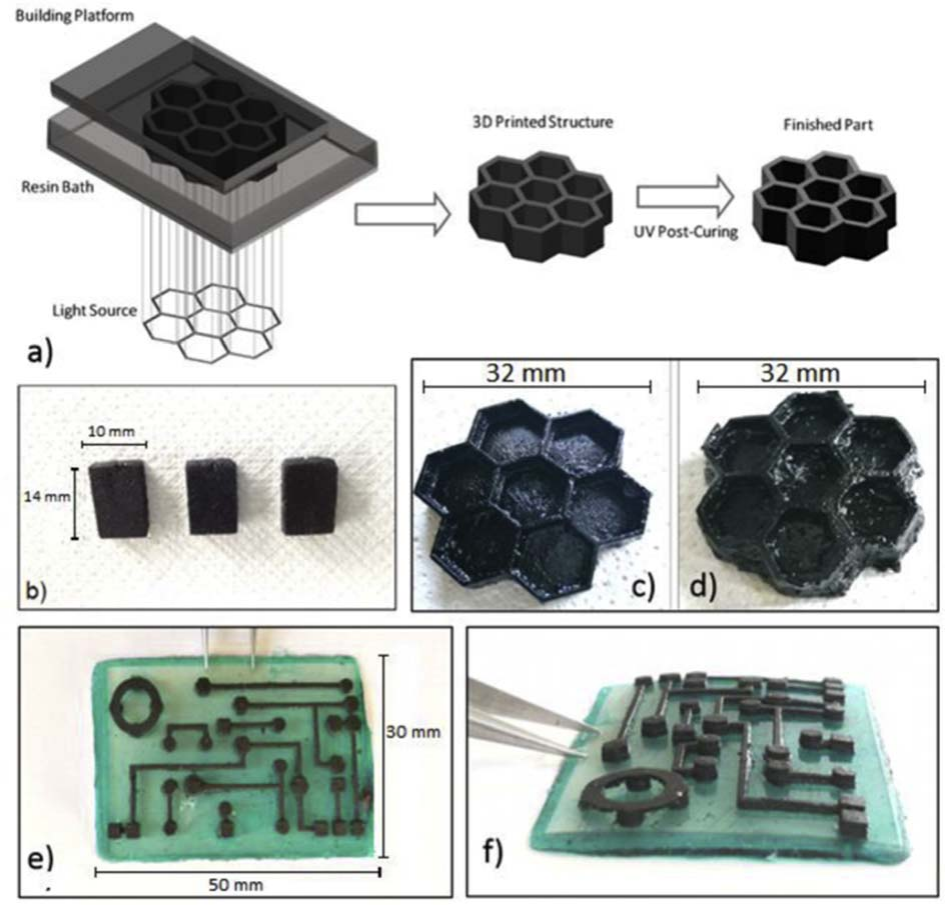
(a) Representative scheme of the DLP 3D printing process. (b) 3D printed cubes (14 × 10 × 3 mm of dimensions) obtained using DLP printer with PEGDA : PEGMEMA 1 : 1.5 wt/wt formulation containing 0.3 wt% CNTs. (c) 3D hexagonal structure (thickness of 5 mm) containing, 0.1 wt% CNTs and (d) 0.3 wt% CNTs. (e and f) Circuit-like structure built on an insulating base (PEGDA : PEGMEMA 1 : 1.5 wt/wt with brilliant green as colorant) with suspended elements containing 0.1 wt% CNTs (reproduced with permission from ref. 148 Copyright 2017, Elsevier).

Gonzalez *et al.* developed a nanocomposite resin using carbon nanotubes (CNTs) with enhanced electrical conductivity. The viscosity and dispersion stability were adjusted by adding a reactive diluent to the polymer matrix. The incorporation of the nanoparticles in the polymer matrix increased the viscosity due to the aggregation and agglomeration of the nanoparticles. A too viscous resin cannot be printed, and therefore proper attention was given to adjust the viscosity. Similarly, with the addition of nanofiller concentration, the glass transition temperature (*T*_g_) decreased, which was because of the reduction in crosslinking density due to the high absorption of the nanofillers. The mechanical properties also decreased by increasing the CNT concentration. When 0.1 wt% CNTs was used, the resulting elastic modulus was 70% higher than that of the control specimens but then it decreased with the addition of CNTs. The reverse trend was found for electrical measurements, as shown in Fig. 27. The addition of 0.1 wt% CNTs enhanced the conductivity by two orders of magnitude compared to the neat specimens and further increased with a high amount of nanofiller.^148^

**Fig. 28:**
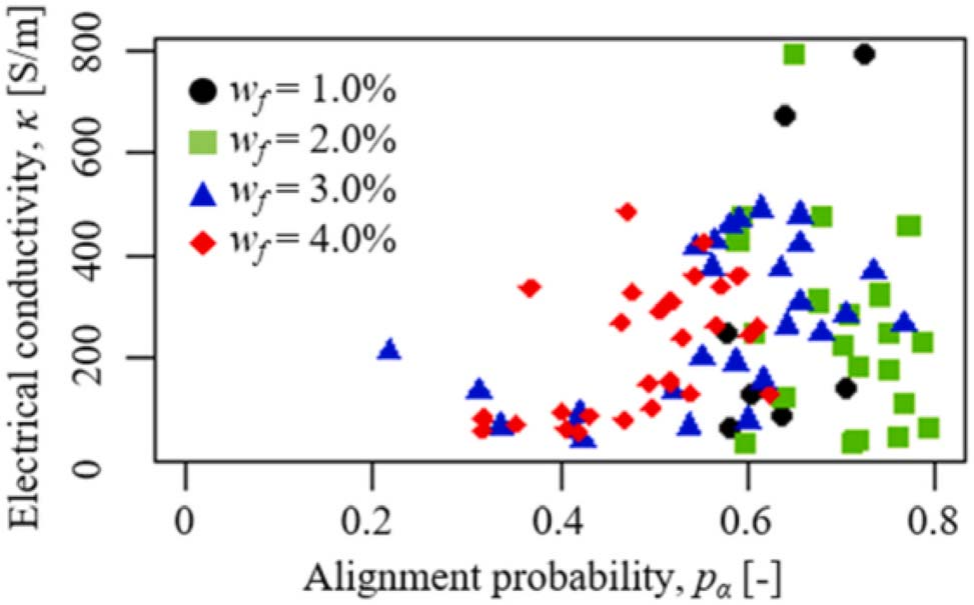
Electrical conductivity, *κ*, as a function of microfiber alignment probability, *p*_α_, for different values of the microfiber weight fraction, wf (reproduced with permission from ref. 138. Copyright 2021, Elsevier).

In another study, Niendorf *et al.* combined SLA 3D printing and the ultrasound-directed self-assembly approach to produce polymer nanocomposites with anisotropic electrical conductivity. The electrical conductivity of the printed specimens was related to different process parameters including transducer power, the filler concentration and alignment as these parameter are critical for a long-range percolated network of conductive fillers. The electrical conductivity of the printed specimens was in the range of 31–793 S m^−1^. The process parameters had a direct effect on conductivity and are critical in predicting the conductive or insulating properties of the samples, as shown in Fig. 28. The practical applications of this approach have some engineering applications such as electromagnetic interface shielding, flexible electronics, and embedded electrical wiring materials.^138^

Scordo *et al.* prepared a high-conductive nanocomposite resin for SLA using poly(ethylene glycol) diacrylate (PEGDA) enriched with the conductive nanofiller poly(3,4-ethylenedioxythiophene):polystyrene sulfonate (PEDOT:PSS). The photoinitiator (PI), surfactant and nanofiller concentration were optimized to get a conductive resin. When the PI concentration was increased up to 6 wt%, the conductivity decreased, and the samples became well-polymerized and solid; however, the nanocomposite resin was very unstable and prone to unwanted polymerization. When the concentration of nanofiller used was 5 wt%, excellent mechanical properties of 21 MPa and remarkable electrical conductivity of 0.05 S cm^−1^ were achieved, thus resulting in a suitable material for use in miniature systems such as microsensors and microfluidic devices. The good mechanical and electrical properties were preserved as the 3D printed materials obtained their structural integrity and conductivity for 30 days.^146^

In another study, Yang *et al.* proposed a polymer nanocomposite resin for the printing of dielectric capacitors by projection-based stereolithography. In this study, PZT nanoparticles were coated with Ag, and then mixed in a photo-curable polymer resin to fabricate dielectric capacitors. The dielectric permittivity was remarkably enhanced by the effective coating and MWS effect. Similarly, the low loss of 0.028 at 100 Hz was attributed to the blockage of charge transfer by the nanocomposite resin and suppression of electric distortion. The maximum specific capacitance achieved was about 63 F g^−1^ at a current density of 0.5 A g^−1^.^152^

**Fig. 29:**
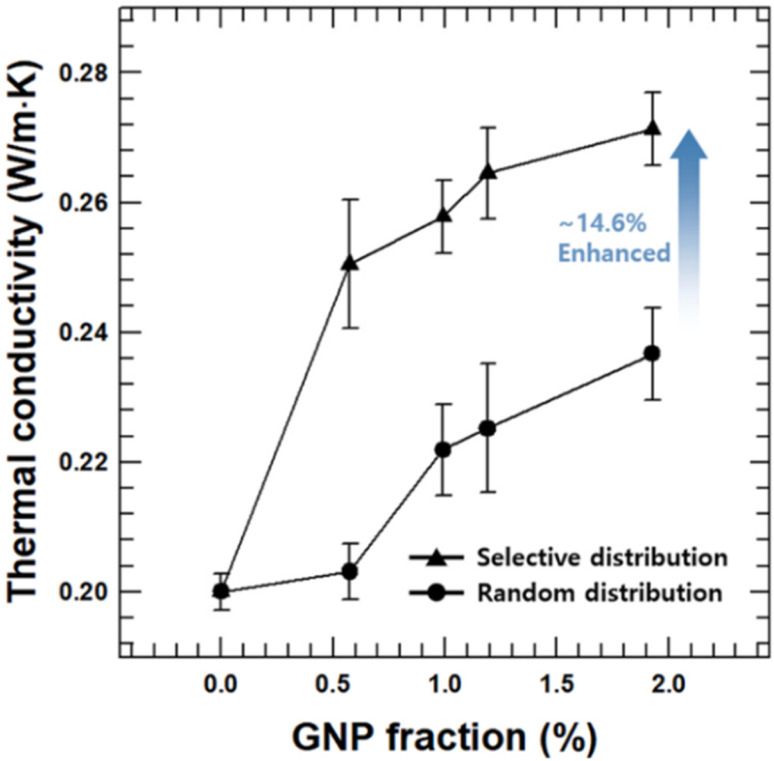
Thermal conductivity of HDDA/epoxy composites with varying amounts of selectively distributed GNP, and epoxy composites with varying amounts of randomly distributed GNP. Selectively distributed GNPs enhanced the conductivity by 14% (reproduced with permission from ref. 144. Copyright, 2021 Elsevier).

Park *et al.* fabricated graphene nanoplatelet (GNP)-based nanocomposites with enhanced thermal conductivity. A commercially available HDDA-based resin was used with some additional additives with varying GNP concentrations (0, 1, 2, 3, 4 wt%). Increasing the GNP concentration in the polymer matrix to a certain extent enhanced the thermal conductivity by 14.6% compared to the random distribution of GNP, as shown in Fig. 29. Greater than 4 wt% GNP concentration reduced the thermal conductivity and mechanical properties as excessive nanoparticles agglomerated, which blocked the light source and resulted in incomplete curing. In comparison with epoxy, the 3D-printed HDDA nanocomposite showed poor performance in mechanical properties with a value of 14.9 MPa.^144^

**Fig. 30:**
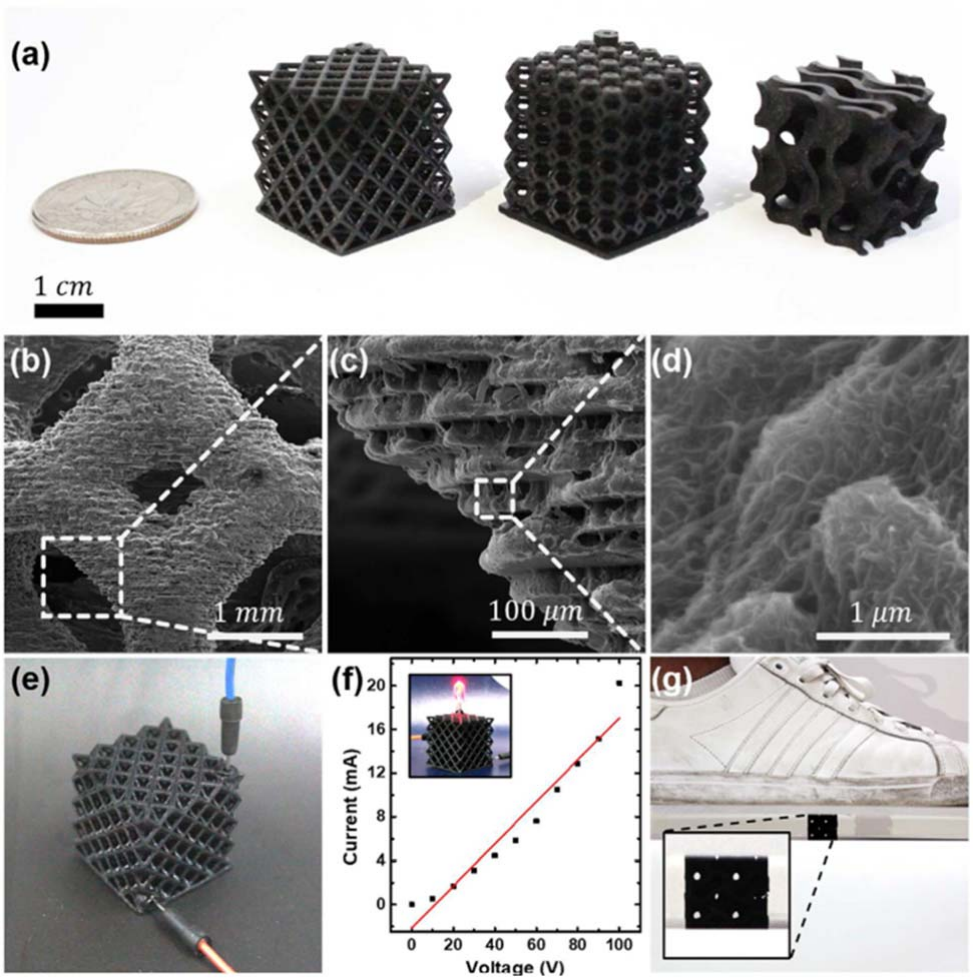
3D-printed conductive complex metamaterial: (a) mechanical metamaterials constructed using 0.6 wt% MWCNT nanocomposites (left), Kelvin foam (center), and gyroid (right). (b–d) SEM images of Kelvin foam with 0.6 wt% MWCNT. (e) Wiring with jumper cables in metamaterial structure. (f) Current *versus* voltage curves from 3D-printed conductive structure. (g) Gyroid structure with mechanical load of up to 70 kg (reproduced with permission from ref. 139. Copyright 2022, Elsevier).

Lim *et al.* improved the electrical conductivity of the resulting nanocomposite by adding multi-walled carbon nanotubes (MWCNT) by solution intercalation method. Given that the efficient dispersion of nanoparticles in the host matrix is critical for customized properties, proper attention was paid to this issue. MWCNTs were dispersed in a polyurethane-based commercial resin using tip sonication, maintaining high dimensional accuracy and good electrical conductivity. Complex structured metamaterials and different capacitive sensors were fabricated from the resulting conductive nanocomposite. It was observed that high dimensional accuracy and electrical conductivity of up to 0.071 S m^−1^ were achieved using a nanofiller concentration of 0.6 wt%, as shown in Fig. 30. This is the first study of its kind to provide a correlation between nanofiller concentration and printing quality by quantitatively measuring the dimensional accuracy.^139^

Wang *et al.* prepared an industrially feasible approach for preparing a UV photo-curable nanocomposite by using cellulose nanocrystals (CNCs) as a nanofiller. The CNCs were grafted to the methacrylate malate photocurable resin to improve their uniform dispersion in the matrix, as shown in Fig. 32. Increasing the CNC concentration up to 1 wt% increased the dielectric constant from 4.0 to 10.9 at 1 MHz. This was attributed to the excellent interfacial effects between the nanoparticles and polymer resin and the presence of abundant (–OH) groups. This prepared nanocomposite resin was used in an energy storage application, where the capacitance of the 1 wt% CNC ring-shaped capacitor improved by 132% compared to the control specimens.^142^

**Fig. 31:**
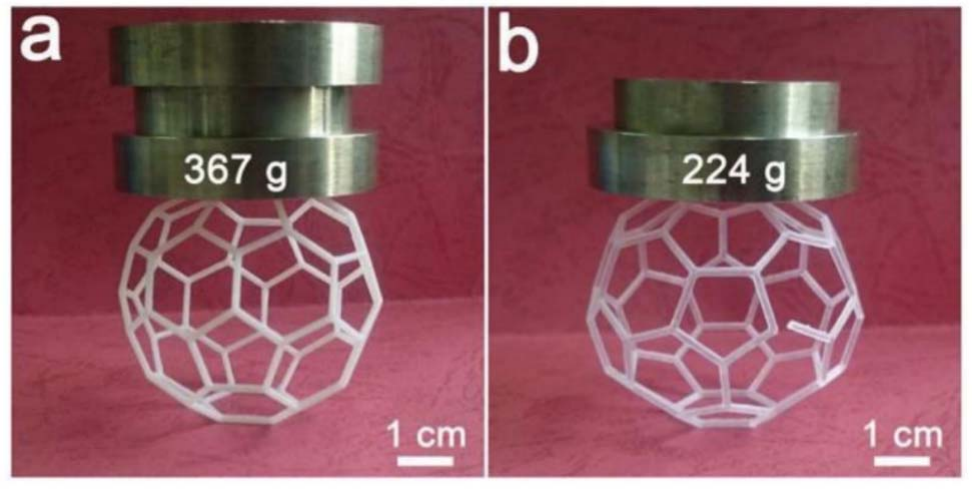
Long-term stability of 3D printed objects by compression test. (a) HDDA resin with β-CEA-AlOOH (4 wt%) and (b) neat HDDA resin. The weights of stainless steel plates on top of model was 367 g (a) and 224 g (b), respectively (reproduced with permission from ref. 137. Copyright 2018, Elsevier).

The thermal conductivity of halloysite nanoclay was found to be greater than that of silver and copper nanoparticles, with the addition of 0.3 wt% and increased from 0.681 W mK^−1^ to 0.721 W mK^−1^ compared to the clear resin.^156^ This increase in conductivity was due to the good interface between the nanofiller and the polymer matrix, which created conductive paths, increasing the conductivity. In a similar study, the electrical conductivity increased by decreasing the nanofiller content and was found to be greater than that of silver nanoparticles. This was because the electrical resistance showed by copper was greater than that of silver nanoparticles.^157^ The long-term stability of boehmite wires improved the mechanical properties of the resulting nanocomposites, as shown in Fig. 31.^137^

**Fig. 32:**
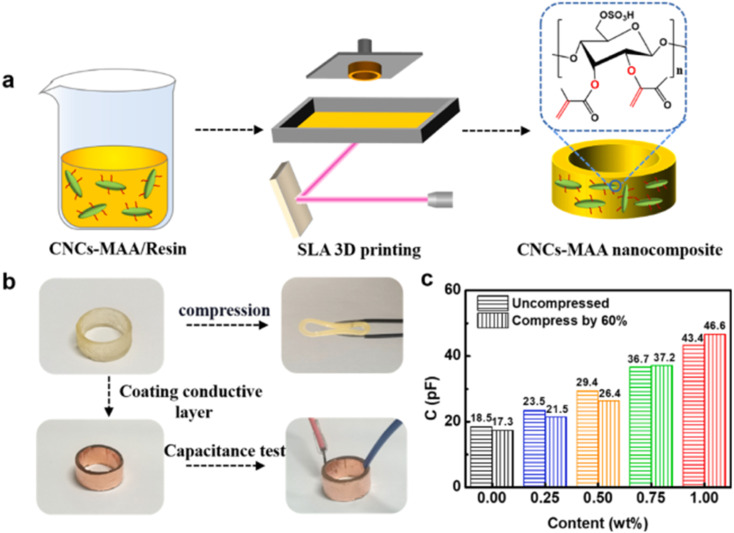
(a) Diagram of 3D printing process of a ring capacitor. (b) Assembly process of a ring capacitor by 3D-printed composites and this printed module had good flexibility. (c) Capacitance without compression (left) and 60% compression (right) at various CNC-MAA contents (reproduced with permission from ref. 142. Copyright 2022, Elsevier).

Post-treatment in the form of thermal curing or UV curing also affects the electrical properties. In fact, thermal curing is more feasible than UV curing in terms of enhancements in electrical conductivity.^60^ In the study by Chiappone *et al.*, the electrical conductivity of 0.3 wt% GO in an acrylate mixture increased from 50.8 nS cm^−1^ to 109.5 nS cm^−1^ by applying thermal post-curing, while the conductivity decreased to 15.72 nS cm^−1^ when it was post-cured by UV treatment.^153^

## 7. Challenges, proposed solutions, and future directions

Despite the many benefits of VPP using nanocomposites, there are several challenges in VPP-based 3D printing when dealing with nanofillers, which hinders the expansion of this technology. The problems that need to be addressed and require further research and development are as follows:

(1) VPP has a greater advantage in terms of resolution, surface quality, design flexibility, *etc.* in comparison to other AM processes but due to the limited material selection of only photopolymer resins, there is a strong need to develop a variety of new materials with excellent properties that are less expensive than current materials.

(2) Viscosity becomes a problem when dealing with photopolymer resins using nanofillers. The incorporation of nanofillers in the photopolymer resin tends to increase in its viscosity, which affects the print process, and consequently affects the desired properties of the printed part. Some photopolymer resins with increased viscosity have high toughness, elasticity, and other desirable properties. However, due to the fact that the current VPP processes cannot print high-viscosity resins, the properties of the photopolymer resins are greatly limited. Therefore, new developments in materials and machines are important to alleviate this issue.

(3) Generally, in the preparation of nanocomposites, the solution intercalation method is applied, which needs a solvent for premixing and filler dispersion. However, the removal of the solvent is difficult and even small traces of solvent molecules can affect the printability and the resulting properties. Some researchers tried a solvent-free approach in the preparation of nanocomposites. Similarly, the aggregation of the nanofillers inside the polymer matrix is also a serious issue, which increases the viscosity, scatters the light, and affects the mechanical properties. Thus, it is necessary to develop enhanced nanocomposite preparation routes to enable the nanofiller to be homogeneously dispersed in the polymer matrix without containing any solvent molecules. In this case, the functionalization of the nanofillers or an optimized sonication route can help reduce agglomeration.

(4) Most nanofillers studied in AM are used to enhance the mechanical properties. However, as there is a lot of variation in mechanical properties by different AM methods, it is not possible to make a single standard for tests and characterizations that is followed by all methods but still there is a need for a set of rules to standardize the mechanical testing/characterizations of 3D-printed polymer parts made by VPP.

(5) The presence of nanofillers in the resin scatters or absorbs the incoming light source at the expense of resolution. Research on new photoinitiators PI and nanofillers is a current requirement to enable photoinitiators to absorb maximum light, while the nanofillers minimize the light scattering or absorbing phenomena.

(6) The utmost aim of any technology or modification in technology is to serve humans. Nanofillers in VPP can help in many ways by enhancing the desired properties. However, nanofillers are not very common in VPP and are mostly limited to lab-scale use as most nanofillers are very expensive and their laboratory preparation requires a skilful and difficult route. Therefore, less expensive nanofillers and photopolymer resins are strongly needed to benefit from the synergy of VPP and nanotechnology.

(7) The integration of nanofillers in VPP involves a lot of complex reactions, different designs, printing parameters, *etc.* Therefore, to fully address these complexities, comprehensive knowledge about the photopolymerization, curing kinetics, light source, design of experiments, *etc.* is required, which will aid successful printing with the desired properties.

## Conflicts of interest

There are no conflicts to declare.

## Supplementary Material
