# Correction: Vat photopolymerization-based 3D printing of polymer nanocomposites: current trends and applications

**DOI:** 10.1039/d3ra90074f

**Published:** 2023-08-14

**Authors:** Mussadiq Shah, Abid Ullah, Kashif Azher, Asif Ur Rehman, Wang Juan, Nizami Aktürk, Celal Sami Tüfekci, Metin U. Salamci

**Affiliations:** a Additive Manufacturing Technologies Application and Research Center-EKTAM Ankara Turkey abid.ullah@gazi.edu.tr abidmech95@gmail.com; b Department of Mechanical Engineering, Faculty of Engineering, Gazi University Ankara Turkey; c State Key Laboratory for Mechanical Behavior of Materials, School of Materials Science and Engineering, Xi'an Jiaotong University P. R. China; d CAS Key Laboratory of Mechanical Behavior and Design of Materials, Department of Modern Mechanics, University of Science and Technology of China P. R. China; e ERMAKSAN Bursa 16065 Turkey; f Department of Industrial Engineering, Nanchang Hangkong University Nanchang P. R. China; g Advanced Manufacturing Technologies Center of Excellence-URTEMM Ankara Turkey

## Abstract

Correction for ‘Vat photopolymerization-based 3D printing of polymer nanocomposites: current trends and applications’ by Mussadiq Shah *et al.*, *RSC Adv.*, 2023, **13**, 1456–1496. https://doi.org/10.1039/D2RA06522C

The authors regret the omission of a funding acknowledgement in the original article. The corrected funding acknowledgement is as shown below.

This work has received financial support from the European Union's Horizon 2020 research and innovation program under the Marie Skłodowska-Curie grant agreement no. 101034425. This work was also supported by the National Natural Science Foundation of China (Grant No. 51974224, 52174372), and the Natural Science Foundation of Shaanxi Province (Grant No. 2020JM-047). This study has also received funding from the Scientific and Technological Research Council of Türkiye (TUBITAK) with grant no. 120C158 for the A2M2TECH project under the TUBITAK's 2236/B program.

The Royal Society of Chemistry apologises for these errors and any consequent inconvenience to authors and readers.

## Supplementary Material

